# Polee: RNA-Seq analysis using approximate likelihood

**DOI:** 10.1093/nargab/lqab046

**Published:** 2021-05-25

**Authors:** Daniel C Jones, Walter L Ruzzo

**Affiliations:** Paul G. Allen School of Computer Science & Engineering, University of Washington, Box 352350, Seattle, WA 98195-2350, USA; Paul G. Allen School of Computer Science & Engineering, University of Washington, Box 352350, Seattle, WA 98195-2350, USA; Department of Genome Sciences, University of Washington, Box 355065, Seattle, WA 98195-5065, USA; Fred Hutchinson Cancer Research Center, 1100 Fairview Ave. N., P.O. Box 19024, Seattle, WA 98109, USA

## Abstract

The analysis of mRNA transcript abundance with RNA-Seq is a central tool in molecular biology research, but often analyses fail to account for the uncertainty in these estimates, which can be significant, especially when trying to disentangle isoforms or duplicated genes. Preserving uncertainty necessitates a full probabilistic model of the all the sequencing reads, which quickly becomes intractable, as experiments can consist of billions of reads. To overcome these limitations, we propose a new method of approximating the likelihood function of a sparse mixture model, using a technique we call the Pólya tree transformation. We demonstrate that substituting this approximation for the real thing achieves most of the benefits with a fraction of the computational costs, leading to more accurate detection of differential transcript expression and transcript coexpression.

## INTRODUCTION

The past decade has seen RNA-Seq become a central tool in molecular biology research. Along the way there have been numerous methods developed to analyze this data. We will propose an entirely new methodology based on likelihood approximation, which enables inference on full probabilistic models that would otherwise quickly grow intractable. In preparation, we will first give a brief overview of notable approaches to RNA-Seq transcript and gene quantification, to give some sense of where this new method fits in.

Gene/transcript quantification is not the only application of RNA-seq. Most prominently, the technology has been used to discover and annotate new transcripts, either by *de novo* assembly (1–3), or processing reads aligned to a reference genome sequence (4–6). Other applications include detection of fusion transcripts (7) and RNA editing (8). We will largely ignore these uses of RNA-seq to focus squarely on quantification. For the most part, we will assume that a suitable reference genome sequence and transcript annotations are available (or simply transcript sequences).

Because of alternative splicing, alternative transcription start and termination sites, and paralogous genes, transcripts often have a degree of sequence similarity that renders short reads ambiguous. Short read RNA sequencing in transcriptionally complex organisms thus produces a mixed signal. A broad distinction that must be drawn among quantification methods is between those that avoid trying to deconvolute these mixed signals and those that embrace deconvolution. A fundamental assumption of most RNA-seq analyses is that transcript expression is proportional in expectation to the number of reads observed from that transcript (when transcript lengths and sample specific effects are accounted for). As Mortazavi *et al.* (9) notes, read counts were observed to be ‘linear across a dynamic range of five orders of magnitude in RNA concentration’. Yet reads of ambiguous origin cannot be trivially assigned to transcripts. Estimating transcript expression thus necessitates either ignoring ambiguous reads, explicitly assigning them to transcripts, or otherwise implicitly considering the space of possible assignments.

Transcript expression is not the only possible quantity of interest. Many methods have found success using different sets of less ambiguous features. The most obvious choice is genes, which are sometimes ambiguous, but far less so than isoforms. Less obvious sub-gene features are also used. DEXSeq (10) approaches the problem by considering exon usage. JunctionSeq (11) and LeafCutter (12) are both methods that specifically focus on reads crossing splice junctions, with the goal of detecting changes in usage. Two recent methods (13,14) both focus on *equivalence classes*, which are sets of transcripts that do not share any reads. Similarly, Yanagi (15) first segments the transcriptome into relatively unambiguous features, before applying a count based analysis.

Finding creative ways to bypass the issue with ambiguous features is a powerful approach that enables analyses that are computationally efficient and statistically powerful. Yet, we should not lose track of the fact that transcript expression is the most direct and interpretable quantity of interest. Interrogating transcript expression necessitates confronting read ambiguity head on. Though there are non-probabilistic approaches to this problem, including methods using network flow for transcript quantification (16) and assembly (17), and integer programming (18), the probabilistic approach has been by far the most common and is the one we will focus on here.

The common probabilistic approach to the transcript quantification problem is to treat transcripts as inducing distinct probability distributions over reads (or read pairs). Starting with the assumption that we have a known transcriptome consisting of *n* annotated transcripts, the experiment as a whole can then be thought of as a mixture model, in which the goal is to infer relative transcript expression (i.e., mixture coefficients). More explicitly, given a set *r* of *m* reads, we define a probability function *p*_*j*_ over possible reads, for every transcript *j* ∈ {1, …, *n*}. The likelihood for a relative expression vector *x* ∈ Δ^*n* − 1^ (here Δ^*n* − 1^ is the open unit (*n* − 1)-simplex, i.e. the set of all vectors of length *n*, with entries in (0,1) summing to 1) is

(1) $$\begin{equation*} P(r|x) = \prod _{i=1}^{m} \sum _{j=1}^{n} x_{j} p_{j}(r_i) \end{equation*}$$

There are many issues surrounding the question of how best to model each transcript read distribution *p*_*j*_. RNA-Seq protocols involve many steps like fragmentation, reverse transcription, amplification, and fragment size-selection which each influence the observed distribution of reads. Assuming reads to be uniformly distributed across a transcript, subject to some fragment length distribution, is the most straightforward model, but some success has been had in building more accurate models that capture positional and sequence-specific biases (see e.g. (19–23)). Though we do account for these effects in practice (see Supplementary Section 6), we will set these issues aside for now and simply assume we have some agreed upon model.

Relative transcript expression for an individual RNA-Seq sample is not typically interesting on its own. RNA-Seq experiments are nearly always concerned with detecting transcriptional changes between groups of samples. From a Bayesian perspective, we would like to build a model of transcriptional changes among *k* samples consisting of sets of reads *r*^(1)^, …, *r*^(*k*)^, with model parameters θ (e.g. effect sizes, pooled means, or latent space encodings), then consider the posterior distribution *P*(θ|*r*^(1)^, …, *r*^(*k*)^)∝*P*(*r*^(1)^, …, *r*^(*k*)^|θ)*P*(θ).

If we adopt the likelihood function in Equation (1), then *r*^(*i*)^ is independent of all other variables when conditioned on *x*^(*i*)^. In typical models expression vectors *x*^(*i*)^ will also be mutually independent when conditioned on the model parameters θ, so we can write this posterior distribution in terms of the latent expression vectors *x*^(1)^, …, *x*^(*k*)^, treating them as nuisance parameters.

(2) $$\begin{eqnarray*} &&P(\theta |r^{(1)},\dots ,r^{(k)}) \nonumber \\ &&\propto \int _{x} P(r^{(1)},\dots ,r^{(k)}|x^{(1)},\dots ,x^{(k)}) P(x^{(1)},\dots ,x^{(k)}|\theta ) P(\theta ) dx \nonumber \\ &&= \int _{x} \prod _{s=1}^{k} P(r^{(s)}|x^{(s)}) P(x^{(s)}|\theta ) P(\theta ) dx \end{eqnarray*}$$

This is all very straightforward but presents some practical problems. To evaluate the likelihood functions, some information about each unique read must be stored (typically in a sparse matrix where entry *i*, *j* corresponds to the probability assigned to the *i*th read by the *j*th transcript distribution). This translates to hundreds of megabytes to several gigabytes per sample. Estimating the posterior for moderately large experiments requires either a great deal of memory or cycling read data in and out of memory, as is done in stochastic gradient methods.

In practice, this kind of textbook model is often short-circuited. Point estimates are made for transcript expression vectors *x*, usually by maximum likelihood

$$\begin{equation*} x^{(i)*} = {arg\, max}_{x^{(i)}} P(r^{(i)}|x^{(i)}) \end{equation*}$$

Then these are plugged into the full model, forming an alternative posterior distribution

$$\begin{equation*} P(\theta |x^{(1)*}, \dots , x^{(k)*}) \end{equation*}$$

This model adopts the (false) assumption that these expression values are observed, substituting them for what is actually observed: the reads. Non-Bayesian models have the same issues, and often resort to the same two-step approach. When estimates are treated as observations, the uncertainty of these estimates is flushed from the analysis, artificially inflating the certainty of the end result. This two-step approach can be thought of as an approximation of the desired model, but one that captures none of the uncertainty of read assignments.

Some methods have been developed to account for this estimation uncertainty. In prior work, we built a joint model that avoids the two step approach, directly conditioning on reads (24). This produced accurate expression estimates but only scaled to a relatively small number of samples. BANDITS (25) uses equivalence class counts as input, then models transcript level counts as observed variables, doing inference on a full model. BitSeq (26) implements a two step model in which transcript expression is sampled using MCMC and these samples used in the second step as ‘pseudo-data’. MMSEQ (27) incorporates MCMC samples by fitting a Normal distribution to the samples and using that distribution in the differential expression model, while Swish (28) introduced a way of using MCMC samples directly in nonparametric tests of differential expression. In later work, variational inference was added to BitSeq (29), but observing an underestimation of variance, the authors express reservations about using it to call differential expression. Using a generalized Dirichlet distribution (30) was shown to help reduce this issue.

In Sleuth (31), variance is estimated from bootstrap samples of maximum likelihood estimates. Incorporating these variance estimates into regression models, they show substantial improvements in accuracy when calling differential expression, particularly at the transcript level. IsoDE2 (32) instead used bootstrap samples to directly compute confidence intervals over fold changes. Bootstrap methods do have limitations. Estimates of variance are guaranteed to converge asymptotically to the true value with enough reads, but this leaves lightly sequenced loci with potentially unreliable estimates.

Whether using the bootstrap or MCMC, generated samples have limited applications. For example, powerful probabilistic programming languages have become an increasingly popular way to implement models, but efficient inference is usually performed using variational inference or some form of Hamiltonian Monte Carlo which rely on directly evaluating and differentiating the likelihood function. Variational methods of approximating the likelihood produce just such a function, but are highly contingent on the distribution family used to make the approximation. An insufficiently flexible family will systematically underestimate variance, inflating false positives.

**Figure 1.**
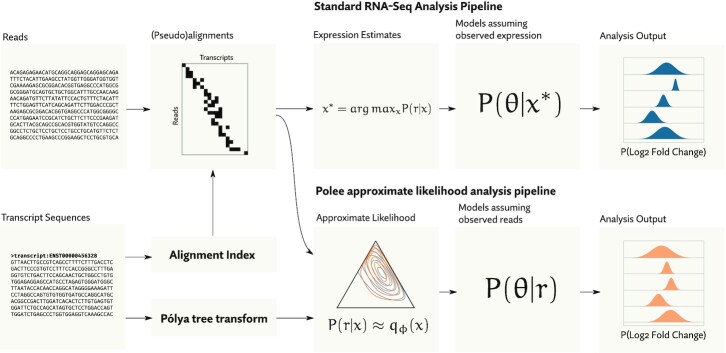
An overview of the proposed analysis pipeline. Standard RNA-Seq pipelines work from intermediate point estimates of expression (x*), then go on to assume those estimates are observed. Our method instead makes an intermediate approximation of the likelihood function using a Pólya tree transformation. Using this in downstream models has the effect making a more realistic assumption: reads are observed. This principled accounting of uncertainty leads to more accurate downstream analysis, especially when features are ambiguous, as is the case with isoform level quantification.

**Figure 2.**
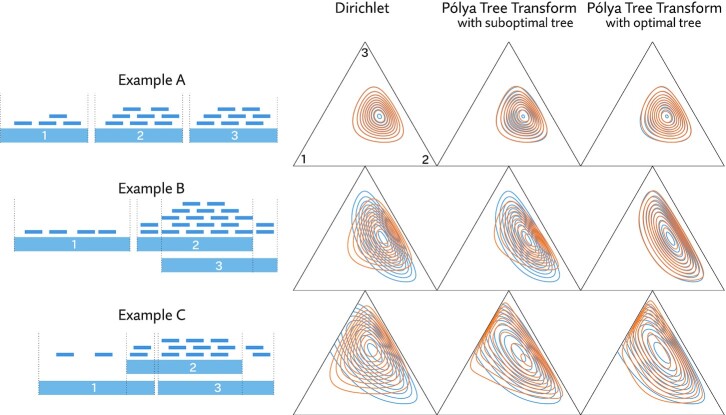
Contrived examples of fitting an RNA-Seq likelihood function. Three examples of hypothetical RNA-Seq reads covering three transcripts are shown on the left. On the right, in blue, contours of the their likelihood functions are plotted, and in orange, various approximations made by minimizing KL divergence. The approximation we propose, based on what we call the Pólya tree transformation, is shown in the second and third columns. This transformation is defined in terms of a tree. Choosing the right tree plays a large role in how well the approximation fits. In example A, every read can be unambiguously attributed to a single transcript. In this easy case, a Dirichlet distribution is perfectly proportional, and the approximation, regardless of the tree, is a near perfect fit. In examples B and C, reads cannot by unambiguously assigned. Here, the proposed approximation remains a good fit, provided the right tree is selected. Using the approximation in place of the exact likelihood function can enable dramatically more efficient inference.

With this in mind, we developed a compact, highly accurate approximation of the likelihood function that can be efficiently evaluated and differentiated. Because the likelihood function for a full experiment factors into per-sample likelihood functions (as in Equation 2), this approximation can be built one sample at a time. Once fit, evaluating and sampling from the approximation is orders of magnitude faster than using the likelihood function. Substituting this approximation for the real thing can make inference on full probabilistic models, with billions of reads, tractable on even modest computers. The basis of this new method in a technique we call the *Pólya tree transformation*, which allows us to fit our approximation to the sparsity structure of the data, avoiding systematic underestimation of variance common to other variational methods. The software we implement to apply this method we call *Polee*, a portmanteau of ‘Pólya tree’. Figure 1 gives an overview of our approach to approximate likelihood, while Figure 2 provides some illustrative examples of fitting an approximation.

There has not been much work exploring the idea of approximating the RNA-Seq likelihood function to facilitate tractable posterior inference, but one notable exception is Zakeri *et al.* (33) who developed an approach in which reads that have similar values assigned by *p*_*j*_( · ) for every transcript *j* are treated as equivalent and combined in Equation (1). This can significantly improve efficiency of the likelihood function with only a moderate decrease in fidelity. What we propose goes further, reducing the likelihood function into an exceedingly efficient constant time and space function, with only slight reductions in accuracy.

## MATERIALS AND METHODS

### Practical benefits of likelihood approximation

Our approach to approximation is unusual: factoring the likelihood, then fitting a proportional approximation to each factor. Though it could be used in other settings, it is particularly well suited for RNA-Seq, compared to other possible approaches.

The obvious alternative for tractable inference is simply to use variational inference on the posterior we are actually interested in. That is, if we have a model of, say, differential expression, with parameters θ, we want to approximate the intractable posterior *P*(θ|*r*). For a large experiment, all of the reads *r* will not fit in memory, but this problem is amenable to stochastic variational inference, or SVI (34). In SVI, batches of data are subsampled to update estimates of ‘local’ latent parameters (transcript expression estimates *x*^(*i*)^ for each sample *i*), before updating ‘global’ latent parameters (θ).

Because SVI algorithms must cycle many gigabytes of sequencing data in and out of memory to repeatedly compute stochastic gradients, they are likely much less efficient than likelihood approximation. Two additional considerations further increase the latter’s desirability.

First is reusability. Large RNA-Seq experiments can be rich with insight, and lend themselves to multiple analyses. Differential expression, differential splicing, clustering, and dimensionality reduction are separate tasks that might be carried out on the same data, each with its own model, each representing a separate inference task. For these tasks, likelihood approximations must be made only once, after which they can be reused over an over. Amortized over every iteration of every analysis typically run on a dataset, likelihood approximation is far more efficient than other approaches to tractable inference.

Second, likelihood approximation can obviate some of the cumbersome data transfer and storage issues with RNA-Seq. High throughput sequencing produces huge datasets, which must be stored and transferred to collaborators, which can mean waiting on long downloads or exchanging hard drives. Our approach to approximated likelihood, on the other hand, summarizes all expression information for a sample using only a few megabytes per sample. The 1443 brain samples produced by the GTEx project (35), are reduced to 7.8GB of likelihood approximation data. The most compact exact representation of the likelihood function for this experiment would be about 1.5TB, often a prohibitively large amount of data to keep in memory.

### Approximating likelihood with variational inference

Variational inference is usually presented as a means of estimating an otherwise intractable posterior distribution. Given a distribution function *p*, and a family of distributions *q*( · ; ϕ) parameterized by ϕ, we fit *q* to *p*, given some data *y*, by choosing ϕ to minimize the Kullback-Leibler (KL) divergence,

$$\begin{equation*} {arg\, min}_{\phi } D_\text{KL}(q(\theta ;\phi )||p(\theta |y)) \end{equation*}$$

A key feature of this method of inference is that *q* does not depend directly on *y*, so that once ϕ is optimized, the approximate probability can be evaluated without the data, only retaining the typically much smaller parameter vector ϕ.

This suggests a solution to the issue of building large joint models. Instead of using variational inference to approximate a posterior distribution, we can use it to separately approximate the likelihood of each sample. By substituting an approximation *q*(*x*; ϕ) for each sample’s likelihood function *P*(*r*|*x*), we can capture the likelihood with some fidelity without having to keep the RNA-Seq reads in memory. This would allow us to build a very large model, encompassing hundreds or thousands of samples, that can be run on laptops or meager servers.

Of course, the likelihood is not a distribution over expression vectors *x* but over reads *r*, so the KL divergence is not well-defined here. But in models making use of the likelihood function, multiplicative constants are generally irrelevant, so our approximation only has to be proportional to the likelihood. To bring the machinery of variational inference to bear, instead of approximating likelihood directly, we approximate a normalized likelihood function, mathematically equivalent to a posterior distribution under a uniform prior. We will denote with $\mathcal {P}(x|r)$ this normalized likelihood function, defined simply as

(3) $$\begin{equation*} \mathcal {P}(x| r) = \frac{P(r|x)}{\int _{x \in \Delta ^{n-1}} P(r|x) } \end{equation*}$$

Revisiting Figure 2 , we can see that capturing the dependence structure of $\mathcal {P}$ requires careful selection of the distribution family being used, but it is often possible to do so very accurately. In simple cases lacking any read ambiguity, the likelihood is proportional to a Dirichlet distribution, but this model is inadequate for cases where reads are compatible with multiple transcripts. The model we propose can perfectly capture the cases of zero read ambiguity (see Supplementary Section 3), but is strictly more expressive, and able to capture more complex dependence structures while being similarly efficient (i.e. linear time and space in the number of transcripts). In this way, it can be thought of as a way of further generalizing the generalized Dirichlet distribution (36). Minimizing the KL divergence is straightforward without need to explicitly normalize the likelihood function (Supplementary Section 4).

Because RNA-Seq data is compositional, our approximating family of distributions *q*(*x*; ϕ) must be defined over a simplex Δ^*n* − 1^. Without going into the mathematical details (see Supplementary Section 3), the common approach to optimizing the KL-divergence additionally requires a distribution that can be expressed as a deterministic bijection of a random variable drawn from some fixed distribution (a technique termed the ‘reparameterization trick’ (37,38)).

The Dirichlet distribution tends to be the default choice distribution over the unit simplex, but besides being insufficiently expressive for our goals (see Figure 2), it is not efficiently computable in terms of reparameterization. Instead, we consider transformations of other distribution families onto the simplex. For this to work we need a bijection $T: \mathbb {R}^{n-1} \rightarrow \Delta ^{n-1}$ (or perhaps *T*: (0, 1)^*n* − 1^ → Δ^*n* − 1^), with an efficiently computable Jacobian determinant.

#### Compositional data analysis transformations

The compositional data analysis literature has traditionally been concerned with how best to transform data to and from the simplex. A number of $\Delta ^{n-1} \rightarrow \mathbb {R}^{n-1}$ transformations have been proposed, some with inverses. When used to transform a normal distribution, for example, these can induce useful simplex distribution families. Three such bijective transformations are explored here as possible candidates to define a suitable distribution.

The first common approach is the additive log-ratio transformation.

(4) $$\begin{equation*} \text{alr}(x) = \left( \log \frac{x_i}{x_n}; i = 1, \dots , n-1 \right) \text{ for } x \in \Delta ^{n-1} \end{equation*}$$

The alr is typically defined, as it is here, with the divisor *x*_*n*_, but the vector can be permuted to make any element the divisor. In some settings it makes sense to choose a particular element as the reference for interpretability; for example we might choose *x*_*n*_ to be the expression of a housekeeping gene. When searching for the best fitting approximation, there is not an obvious choice. If *y* ∼ Normal(μ, Σ), then the distribution induced by alr^−1^(*y*) is sometimes referred to as a *multivariate logit-normal distribution*.

The second useful transformation is the *multiplicative log-ratio transformation* (39)

(5) $$\begin{equation*} \text{mlr}(x) = \left( \log \frac{x_i}{1 - \sum _{j=1}^{i} x_j}; i = 1, \dots , n-1 \right) \text{ for } x \in \Delta ^{n-1} \end{equation*}$$

This transformation can be best understood using a sequential stick-breaking metaphor. If a stick is broken into *n* pieces, and *x*_1_, …, *x*_*n*_ give the size of each piece, in proportion to the whole, then mlr(*x*) gives the log-ratio between each piece and the remaining length of the stick, if the stick were broken one piece at a time. We will return to this stick-breaking metaphor shortly.

The probabilistic programming language Stan (40) implements essentially this transformation as a general purpose variational approximation to distributions on the simplex as part of its Automatic Differentiation Variational Inference approach (41).

The third and most modern approach comes from Aitchison geometry (42). Under specific definitions of scalar multiplication, vector addition, and inner product the simplex forms a inner product space. The *isometric log-ratio* (ilr) transformation (43) is a bijection that maps a point on a simplex Δ^*n*^ to its coordinates in $\mathbb {R}^n$ with respect to some basis. Though it is not necessarily clear how to choose simplex basis vectors for the transformation, one proposed scheme computes vectors corresponding to a full binary tree (44), bearing some resemblance to the transformation we will propose.

**Figure 3.**
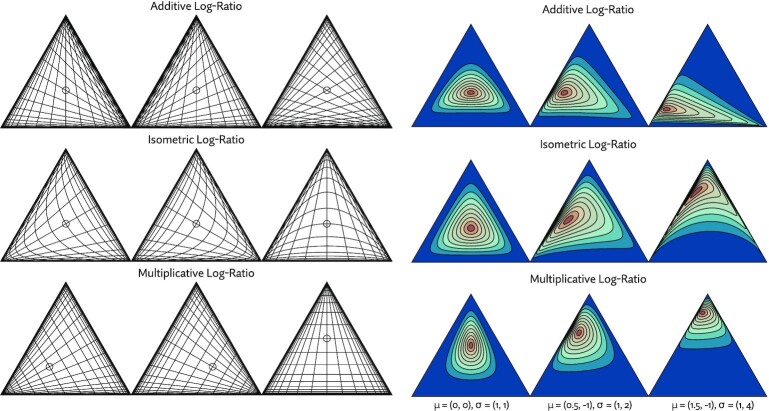
Examples of simplex transformations and simplex distributions that can be induced by transforming a normal distribution. On the left, Cartesian grid lines are transformed onto Δ^2^ using three classes of transformations: additive log-ratio (alr), isometric log-ratio (ilr), and multiplicative log-ratio (mlr). Grid lines are spaced evenly at a distance of 0.5 in Euclidean space and the point (0,0) is marked with a circle. Each transformation has variations shown in the columns. The variations are formed by choosing a different denominator, basis, or permutation for alr, ilr and stick breaking, respectively. On the right, various parameterizations of a 2D multivariate normal distribution, with a diagonal covariance matrix, are mapped onto the simplex with alr, ilr, and mlr (for each, the furthest right of the three variants shown on the left). The choice of transformation has a dramatic effect on the resulting distribution.

Figure 3 gives some intuition for how these transformations operate.

#### The Pólya tree transformation

To revisit the stick-breaking metaphor, it is often easiest to consider generating a vector *x* ∈ Δ^*n* − 1^ by starting with a stick of length 1 and breaking it *n* − 1 times in sequence.

Let *y*_*i*_ ∈ (0, 1) represent the proportion of the remaining stick to break off on the *i*th break. Then *x* ∈ Δ^*n* − 1^ can be produced from *y* ∈ (0, 1)^*n* − 1^ with

(6) $$\begin{eqnarray*} x_i = y_i \prod _{k=1}^{i-1} (1 - y_k) = y_i \left(1 - \sum _{k=1}^{i-1} x_k \right) \;\forall \; 1 \le i < n \end{eqnarray*}$$

(7) $$\begin{eqnarray*} x_n = \prod _{k=1}^{n-1} (1 - y_i) = 1 - \sum _{k=1}^{n-1} x_k \end{eqnarray*}$$

The equivalence between the product and sum form shown here may not be immediately obvious, but is easy to show by induction (45), or by noting that $1-\sum _{k=1}^{i-1} x_k$ is the length of the stick remaining after *i* − 1 breaks.

Stick-breaking metaphors like the one used to describe the mlr transformation have a long history. An early example (45) explores a stick breaking process of ‘random alms’ in which each *y*_*i*_ is uniformly distributed in (0,1) using the motivation of distributing a pile of gold dust to a countably infinite sequence of beggars. The possibility of *y*_*i*_ being drawn independently from arbitrary distributions is also briefly considered. In recent literature, stick breaking occurs most commonly in descriptions of the Dirichlet process, which can be formulated as an infinite stick breaking procedure in which the breaks *y*_*i*_ are Beta distributed variables (46). Random alms can be seen as special case of this where the splits are *U*(0, 1) = Beta(1, 1) distributed. The resulting stick sizes are then used to weight draws from a base distribution. When *n* → ∞ in Equations (6) and (7), and each *y*_*i*_ is i.i.d. Beta(1, θ) distributed, for some θ, the resulting distribution family is commonly denoted GEM(θ), after Griffiths, Engen, and McCloskey (47). This notion of a stick breaking prior been generalized to, among other things, include finite stick breaking distributions (48).

Khan, et al. (49) brings up an issue often ignored in these sequential stick-breaking procedures. They point out that the model represents a kind of decision boundary between every category *i* and the *n* − *i* categories that follow it in the process, so a particular ordering may fit the data poorly if no such boundary naturally exists. Zhang *et al.* (50) take up this issue in a more serious way, demonstrating classification problems with as few as three categories that show dramatic differences in performance depending the permutation of those categories in the stick breaking process. They go on to propose models in which category permutations are inferred along with regression coefficients when performing multinomial logistic regression.

The second key insight that is sometimes neglected is that there are other ways of breaking a stick. Rather than breaking pieces off the stick and setting them aside, we might keep and recursively break both of the resulting pieces. This can be thought of as *hierarchical stick breaking*, as opposed to common *sequential stick breaking*. To define a transformation onto Δ^*n* − 1^, we must always end up with *n* pieces, so *n* − 1 total breaks must still be made. Under these restrictions, breaks in a hierarchical stick-breaking scheme must occur according to a full binary tree with *n* leaves (i.e. where every node is either a leaf or has two children).

With these two insights, we have a space of possible stick-breaking transformations along the lines of Aitchison’s mlr, but considering not just the *n*! permutations of the stick breaking process, but also the $C_{n-1} = \frac{1}{n} \binom{2(n-1)}{n-1}$ (the (*n* − 1)st Catalan number) possible full binary trees with *n* leaves, resulting in a family of $C_{n-1} n! = \frac{(2n-2)!}{(n-1)!}$ possible transformations.

**Figure 4.**
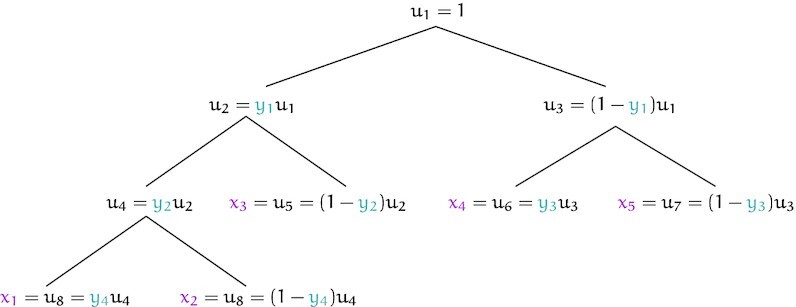
Any example showing the calculations done by the Pólya tree transformation, here taking a vector *y* ∈ (0, 1)^4^ to a vector *x* ∈ Δ^4^. At each node, we compute an ‘intermediate value’ *u*_*i*_ which can be thought of as a stick piece. We recursively ‘break’ these pieces by splitting them according to the elements of *y*. The resulting pieces in the leaf nodes are of positive length and sum to 1, and thus represent a point in the simplex. Importantly, this transformation in a bijection with simple Jacobian determinant. Different tree topologies yield different transformations that correspond to different independence structures.

This notion of hierarchical stick-breaking bears some resemblance to the hierarchical softmax transformation (51), a technique used in some natural language models, but differs critically in that hierarchical softmax is not bijective and is used purely as a means of accelerating inference. More closely related are Pólya tree distributions (52–54), which are also defined in terms of a (not necessarily finite) binary tree, in which each split or break is drawn from a Beta distribution. Due to this similarity, and apparently lacking any existing terminology, we refer to this family of hierarchical stick-breaking transformations as *Pólya tree transformations*. One special case of distribution families induced by Pólya tree transformations is the Hyper-Dirichlet Type 1 distribution described by Dennis (55). Figure 4 shows an example of calculating a Pólya tree transformation on a small vector.

#### Tree topology heuristics

Though Zhang *et al.* (50) were able to effectively optimize over stick-breaking topologies, at most 11 labels were used, and only permutations were considered. We would have a much harder time sampling over the possible configurations of a Pólya tree transformation. To avoid an exhaustive exploration of the space of trees we must either find an adequate heuristic with which to choose a tree, prior to optimizing parameters, or pursue optimization metaheuristics (e.g. simulated annealing or genetic programming) that could explore a subset of the space in a guided way. The latter may be possible, but each topology is accompanied with an entirely new set of parameters that must be optimized. Optimizing both concurrently is unlikely to be practical.

Fortunately, there are reasonable approaches we may take to choosing a topology ahead of optimization. RNA-Seq is typically a sparse mixture model. Most reads are compatible with only a small number of annotated transcripts. Because of this, the problem displays subcompositional independence: knowing the mixture of isoforms expressed in one gene tells us nothing about the mixture of isoforms expressed in another, if the genes share no reads. This suggests that the transformation might be oriented in a way to try to capture this structure.

Aitchison (39) discusses the concept of subcompositional independence, as well as several other notions of independence on the simplex. There, tests for independence are proposed, but they necessitate estimating the full covariance matrix, an intractable task for a large *n*. Instead we pursue the idea of capturing a similar subcompositional independence structure by using hierarchical clustering as a heuristic. Each transcript is represented by its set of compatible reads. We then cluster greedily, choosing the maximum Jaccard index (i.e. the size of the intersection divided by the size of the union) at each step. Transcripts, or sets of transcripts, that share a large proportion of their compatible reads have a higher Jaccard index, and thus their common ancestor is placed lower in the tree. In an ideal scenario, this constructs a tree that encodes a distribution family that has a similar independence structure to that of the real likelihood function.

In cases of complete subcompositional independence, where no reads are shared between transcripts, the likelihood function in Equation (1) is proportional to a Dirichlet distribution. In Supplementary Section 3 we show that any Pólya tree transformation can exactly fit any Dirichlet distribution of the same dimensionality, if applied to appropriately chosen Beta distributed random variables.

#### Choosing a base distribution

Given a transformation onto the simplex, we now need to choose the distribution that will be transformed. The Pólya tree transform has been described here as a (0, 1)^*n* − 1^ → Δ^*n* − 1^ transformation. Most of the compositional data analysis transforms take the form $\mathbb {R}^{n-1} \rightarrow \Delta ^{n-1}$. We can always use the logit function (or its inverse) to move between the two so this distinction is insignificant. We would like then to find a distribution over $\mathbb {R}^{n-1}$ or (0, 1)^*n* − 1^. To minimize the parameter space, each element will be considered independent (a common approach refereed to as ‘mean field variational inference’).

We are limited to distributions that lend themselves to the reparameterization trick. The two distributions we will consider are the normal (or logit-normal) distribution and the Kumaraswamy distribution (56), which is qualitatively similar to the Beta distribution but, unlike the Beta distribution, can be easily expressed as a transform of a uniform distribution.

Lastly we explore a further transformation of the normal distribution using a parameterized sinh-arcsinh transformation of the following form

$$\begin{equation*} U(Z; \alpha ) = \text{sinh}(\alpha + \text{arcsinh}(Z)) \end{equation*}$$

This technique, explored by (57) along with a two-parameter version, provides an analytically convenient way to add a parameter controlling skewness to a distribution. Where *Z* ∼ Normal(0, 1), we use

$$\begin{equation*} Y = \mu + \sigma U(Z; \alpha ) \end{equation*}$$

as our fully reparameterized distribution. We will refer to the resulting distribution as a *skew-normal* distribution, though other distribution families also go by this name (58,59).

### Mathematical details of the Pólya tree transformation

Earlier, we described the Pólya tree transformation as a stick breaking transformation between (0, 1)^*n* − 1^ and Δ^*n* − 1^. Here, we give a more formal definition, and generalize it somewhat by not assuming the composition sums to 1, allowing the stick being broken to be of arbitrary length. We can then think of the transformation as between $(\mathbb {R}_{+}, (0,1)^{n-1})$ and $\mathbb {R}_{+}^{n}$, where $\mathbb {R}_{+}$ is the set of positive real numbers. This can be thought of as mapping an initial stick length, and *n* − 1 break points to *n* stick pieces of positive length. We also state some properties of the transformation, with proofs left to the supplement (Supplementary Section 3).

The Pólya tree transformation is best represented as a full binary tree, with 2*n* − 1 nodes, *n* of which are leaves. To simplify notation somewhat, assume these are assigned indices so that the root node is labeled 1, internal nodes 1, …, *n* − 1, and leaf nodes *n*, …, 2*n* − 1. Additionally, we assume internal nodes are numbered so that no node has a smaller index that any of its ancestors, for example, according to a pre-order traversal. Intuitively, we can think of the *i*th node as representing the *i*th break in a stick-breaking process.

The tree can then be defined by functions giving an internal node’s left and right children respectively.

left(*i*): index of node *i*’s left child
right(*i*): index of node *i*’s right child

The transformation *T* from $(u_1, y) \in (\mathbb {R}_{+}, (0,1)^{n-1})$ to $x \in \mathbb {R}^{n}_{+}$ is defined in terms of *intermediate valuesu*_1_, …, *u*_2*n* − 1_ for each node, which have the following relation,

(8) $$\begin{equation*} u_{\text{left}(i)} = y_i u_i \end{equation*}$$

(9) $$\begin{equation*} u_{\text{right}(i)} = (1 - y_i) u_i \end{equation*}$$

The result of the transformation is then simply the intermediate values from the leaf nodes:

$$\begin{eqnarray*} x_i = u_{i+n-1}, \text{ for } i = 1, \dots , n \end{eqnarray*}$$

Often we are operating on the unit simplex, in which case *u*_1_ = 1. In these instances, we will leave the *u*_1_ = 1 implicit and write the transformation as *T*: (0, 1)^*n* − 1^↦Δ^*n* − 1^. Figure 4 gives an example of of the transformation from (0, 1)^4^ to Δ^4^.

When implemented, *u*_*i*_ values are computed by traversing the tree with any top-down traversal from the root. In the stick-breaking metaphor, intermediate values can be thought of as sizes of intermediate sticks after some number of breaks are performed. The inverse transformation can be computed by traversing the tree up from its leaves, as is done in a post-order traversal.

#### Properties of the Pólya tree transformation

The Pólya tree transformation has a few interesting properties, which we summarize here with proofs and further exposition left to the supplement.

First, importantly for the transformation to be tractable, the Jacobian determinant has a simple form that can be computed in time *O*(*n*). It is simply the product of the internal node intermediate values.

Theorem. *Let* $(u_1, y) \in (\mathbb {R}_{+}, (0,1)^{n-1})$ , *andJ*_*T*_*be the Jacobian matrix ofT*((*u*_1_, *y*)). *Further, letu*_*i*_ for *i* = 2, …, 2*n* − 1 *be the intermediate values as defined in Equations* (8) *and* (9)*. Then*

$$\begin{equation*} |\det (J_T)| = \prod _{i=1}^{n-1} u_i \end{equation*}$$

The proof to this theorem is under Supplemental Theorem 3.4.

The choice of base distribution induces different families of distributions on the simplex under the transformation. If we use Beta distributions as our base and parameterize them by assigning a vector of intensities α to the leaves, then choose Beta parameters to be the sums of the left and right subtree intensities, respectively, we have a class of distributions we call HierarchicalBeta. We go on to show this family is equivalent to the Dirichlet distribution, regardless of the tree topology defining the transformation.

Theorem. *For any Pólya tree transformation* *T*: (0, 1)^*n* − 1^↦Δ^*n* − 1^*wheren* ≥ 2*and any*$\alpha \in \mathbb {R}_{+}^{n}$

$$\begin{equation*} \text{Dirichlet}(\alpha ) = \text{HierarchicalBeta}(T, \alpha ) \end{equation*}$$

The proof to this theorem is under Supplemental Theorem 3.10.

This equivalency opens up the possibility of using Beta distributions as the base distribution, which gives an exact fit in cases of complete subcompositional independence, while generalizing to capture uncertainty information in ambiguous features. This relationship could also be exploited to speed up inference in models with Dirichlet priors.

## RESULTS

To demonstrate the usefulness of our approach to likelihood approximation, we undertook five analyses. In the first two subsections we show that the approximation is a good fit to the likelihood function of actual RNA-Seq datasets, and that it can be efficiently fit and evaluated. Then we focus on demonstrating that it can improve the detection of differentially expressed transcripts, using an existing simulation benchmark, and then separately using real data from the GTEx project. Finally, we show that using the approximate likelihood produces more reliable estimates of of pairwise correlation.

### The Pólya tree transform improves the goodness of fit to likelihood marginals

We explored the fit of a number of possible approximations to the RNA-Seq likelihood function produced transforming standard probability distributions. We used a three bijective transformations from the compositional data analysis literature: additive log ratios (alr), multiplicative log ratios (mlr), (39) and isometric log-ratios (ilr) (43). The transformation we propose, the Pólya tree transformation, is a fully generalized stick-breaking transformation, which can be thought of as an extension of mlr. The transformation is defined in terms of a tree. We evaluated three heuristics for choosing this tree: sequential trees (equivalent to mlr), random trees, and trees based of hierarchical clustering. We also evaluated three possible base distributions to be transformed: the Kumaraswamy (56), logit-normal, and logit-skew-normal distributions. These transformations and tree heuristics are described in detail in the methods section.

We first evaluated the fit of transcript marginal densities using Wilcoxon signed-rank tests. For every transcript in a set of annotations, 1000 samples were drawn from a Gibbs sampler (representing the exact likelihood function) and the same number of samples were drawn from the approximated likelihood. The signed-rank test was run for each transcript, producing a *P*-value.

The Gibbs sampler used 8 randomly initialized chains. Each was burned-in for 2000 iterations, then 25 000 samples were generated and every 25th was saved for analysis, producing a total of 1000 samples. We found these settings sufficient for marginal distributions to have converged for a vast majority of transcripts, where convergence was measured by comparing within-chain and between-chain variance (60).

**Figure 5.**
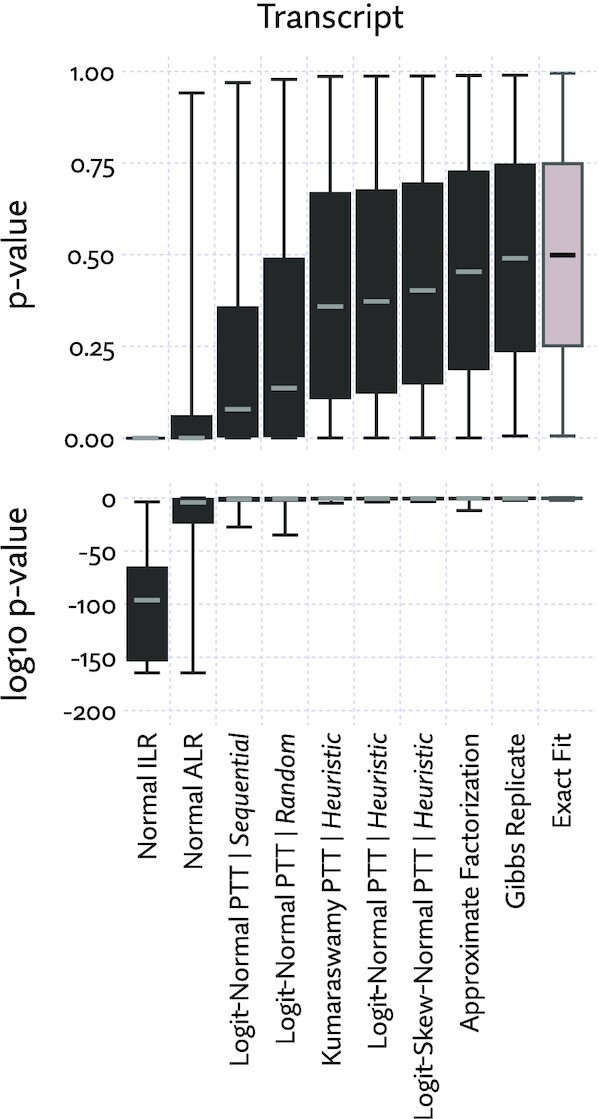
The goodness of fit of several approximations evaluated by inspecting the distribution of *P*-values, where perfect fit would produce a uniform distribution. *P*-values represent the degree of disagreement between 1000 samples from the Gibbs sampler and 1000 samples drawn directly from various approximations of the likelihood function for 16 mouse tissue samples taken from (61). ‘Gibbs Replicate’ is a repeated run of the Gibbs sampler, which is close to a hypothetical exact fit. *P*-values were computed using the Wilcoxon signed-rank test. Here, ‘PTT’ is the Pólya tree transform, which was tried with several distribution families and tree building rules (‘sequential’, ‘random’, and hierarchical clustering, labeled ‘heuristic’). The ilr and alr transformations are defined in the Methods section, and ‘approximate factorization’ is the approximation scheme proposed by Zakeri *et al.* (33). Boxplots are drawn with upper and lower whiskers corresponding to the 99th and 1st percentile, respectively.

**Figure 6.**
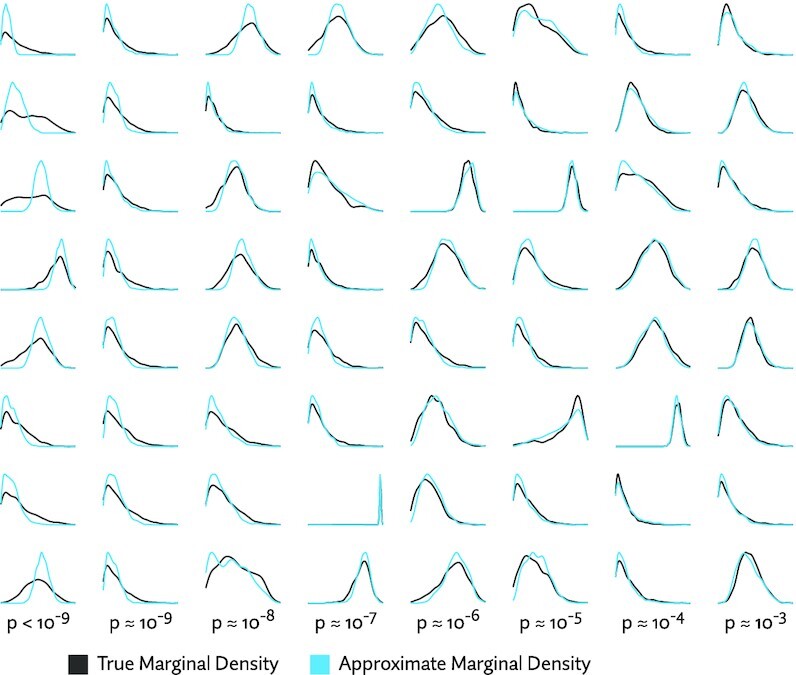
Kernel density plots of exact and approximate marginal densities for examples with small *P*-values. Columns correspond to *P*-values of decreasing powers of 10, while rows are randomly selected examples with approximately that *P*-value, giving a sense of how *P*-value corresponds to goodness of fit. It can be seen that a very small *P*-value does not necessarily correspond to a catastrophic failure of the approximation. The plots shown were generated from a mouse liver sample from (61) with approximate densities using logit-skew-normal Pólya tree transform distribution, using the hierarchical clustering heuristic to choose the tree topology.

If an approximation is a perfect fit, we would expect to see a uniform distribution of *P*-values. Imperfect approximations will yield *P*-value distributions that are increasingly skewed towards smaller numbers. The greater the tendency towards small *P*-values, the worse the overall fit. Figure 5 shows the combined results of this test using a sample from each of 16 tissues taken from a mouse body map data set (61) (accession number PRJNA375882), and 138 930 transcripts from the Ensembl 95 annotations (62). To provide some intuition of the correspondence between *P*-value and fit, a number of examples with low *P*-values are plotted in Figure 6.

We see that the approximating distribution family matters a great deal. Distributions based on the Pólya tree transformation (labeled ‘PTT’) offer a dramatically better fit than the traditional compositional data analysis transforms alr and ilr, and we see that the heuristic tree construction is a sharp improvement over a random tree topology. The common sequential stick-breaking approach (equivalent to the mlr transformation) is seen to be the worst approach to stick-breaking, perhaps in part because such a long chain of dependent breaks is numerically less stable and thus more difficult to fit to the target likelihood. As no attempt was made to optimize over permutations, it is also possible sequential stick breaking could be improved with the right permutation heuristic.

On this test, the approximate factorization approach proposed by Zakeri *et al.* (33) also performs very well, offering a slight median improvement over the Pólya tree transformation. Though far more efficient in time and space that the un-approximated likelihood function, it does not match the low constant time and space of the Pólya tree transformation (Supplementary Figure S2). Exact factorization, however, has little performance benefit as relatively few read pairs will have identical probabilities when complex bias modeling is used.

This results holds across the other samples from the mouse body map data (Supplementary Section 2), but to ensure that the approximation is broadly effective and not somehow tuned to these particular datasets, we also evaluated logit-skew-normal Pólya tree transform distribution with heuristic tree topologies on a variety of of other RNA-Seq datasets, spanning a number of species with varying transcriptional complexity. We see that likelihood approximation has remarkably consistent performance across these samples (Table 1). Curiously though, the approximation appears to fit moderately better with a larger number of transcripts. The median *P*-value for both the human and mouse samples is approximately 0.4, and for the yeast sample, 0.28. Likely this is due to a smaller proportion of the transcripts being expressed in species with extensive alternative splicing.

**Table 1.** Auxiliary datasets used to evaluate the consistency of likelihood approximation performance, as was done in Figure 5. Median *P*-values are all for the proposed approximation (labeled ‘Logit-Skew-Normal PTT|Heuristic’ in Figure 5). A uniform distribution of *P*-values, and thus a median *P*-value near 0.5 is desirable as it would result from an exact fit. Numbers of transcripts listed are those annotated in version 90 of Ensembl. Accession numbers for these samples, from top to bottom, are: SRR453566, SRR030231, SRR065719, SRR023546 and SRR896663

Species	Num. Transcripts	Med. *P*-value
*S. cerevisiae*	7126	0.28
*D. melanogaster*	34 749	0.37
*C. elegans*	58 941	0.35
*M. musculus*	131 195	0.40
*H. sapiens*	200 310	0.40

We also evaluated how approximations affect the estimation of effect size tail probabilities. We define minimum log_2_ fold-change as 90% posterior lower bound on log_2_ fold change. More precisely, if $x^{(a)}_i, x^{(b)}_i$ are the unobserved transcript expression values for transcript *i* from samples *a* and *b*, respectively, then the minimum log_2_ fold change is a number δ_*i*_ where

$$\begin{equation*} P\left(\left| \log _2 \frac{x^{(a)}_i}{x^{(b)}_j} \right| >\delta _i \right) = 0.9 \end{equation*}$$

Minimum log_2_ fold-change is a convenient way of summarizing evidence for differential expression, so error introduced into these estimates by an approximation would be a major concern.

**Figure 7.**
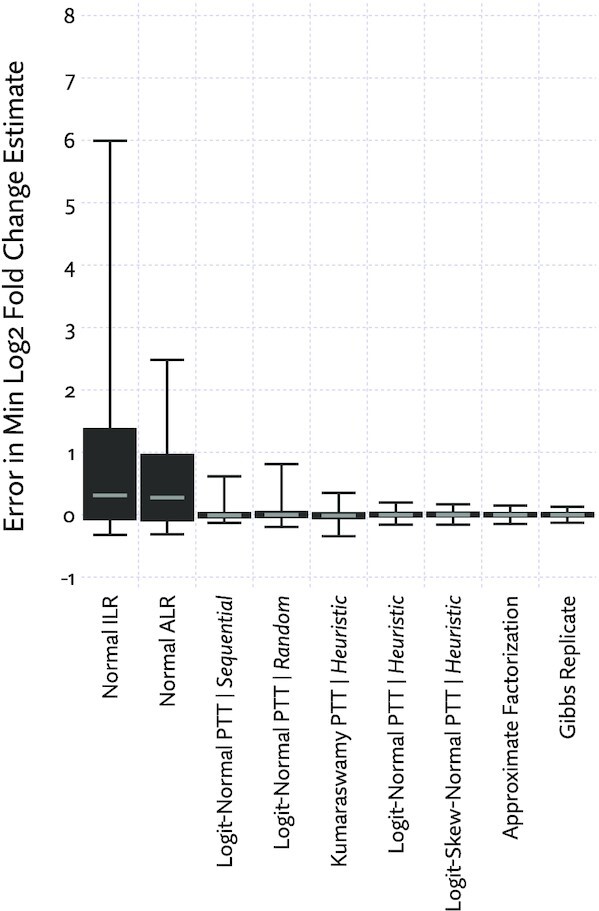
Error in estimates of minimum log_2_ fold-change introduced by various approximations. A positive error indicates an overestimation of the true effect size, potentially introducing false positives, a negative error an underestimation, potentially introducing false negatives. ‘Minimum log_2_ effect size’ is the effect size at which there is a 90% posterior probability that the true effect size is higher. Boxplots are drawn with upper and lower whiskers corresponding to the 99th and 1st percentile, respectively.

We compared estimates of minimum log_2_ fold-change across all pairwise comparisons between individual samples from each of the 16 tissues. The results in Figure 7 broadly agree with the *P*-value goodness of fit tests. The approximate factorization scheme introduces only slightly more error than simply re-running the Gibbs sampler, and the best Pólya tree transformation based approximation only slightly more than that, while being far more efficient. Approximations based on standard compositional data analysis techniques can catastrophically inflate the estimates of fold-change and thus false-positive rates.

### Estimating and sampling from approximated likelihood can be faster than bootstrap sampling

The procedure for sampling expression vectors from our approximated likelihood function is to simply generate a random vector from a Normal(0, *I*) distribution, and apply the transform, which is *O*(*n*) where *n* is the number of annotated transcripts. Because samples are so cheap to generate, for a sufficiently large numbers of samples, it outperforms not only MCMC approaches, but also the extremely fast bootstrap approach implemented in Kallisto (63).

To locate the break even point, we recorded the overall time needed to generate increasingly large numbers of samples with both methods. In both approaches these times include the necessary initialization time. Kallisto uses its own pseudoalignment algorithm, while Polee takes as input existing alignments. To compare on equal ground, we exported alignments generated by Kallisto and used them as input into Polee. Added to the Polee timings is the time Kallisto took to generate these alignments, the time it took to approximate the likelihood function, and to generated the requested number of samples. Both methods were run on 8 CPU cores. We found that past about 100 samples (where each sample is a vector of transcript expression estimates), the amortized cost of sampling becomes less for Polee than Kallisto (Supplementary Figure S1).

Approximate likelihood functions can be evaluated directly, so there is not necessarily a need to sample, but sampling is useful in some applications. For example, Polee can be used as a drop-in replacement for Kallisto when using Sleuth (31) to call differential expression. Speed will be similar or faster, and this sampling technique is not subject to the limitations of bootstrap sampling, which can yield imprecise results for transcripts with a very small number of reads.

### Approximate likelihood models outperform other models in identifying differentially expressed transcripts

We expanded an analysis performed in Pimentel *et al.* (31), which demonstrated superior performance when using Sleuth to call differential expression, especially at the transcript level, using simulated data. We include the likelihood approximation method described here in two ways. First we developed a Bayesian regression model (see Supplementary Section 5) in TensorFlow (64) that makes use of approximated likelihood functions directly, which is labeled ‘polee’ in the results. Second, we generated samples from the approximated likelihood to mimic the output of Kallisto, and used this as input to Sleuth. This approach is labeled ‘polee/sleuth’. With low numbers of replicates Swish will tend to produce low resolution p-values. To compensate, we broke p-value ties using fold-change estimates.

**Figure 8.**
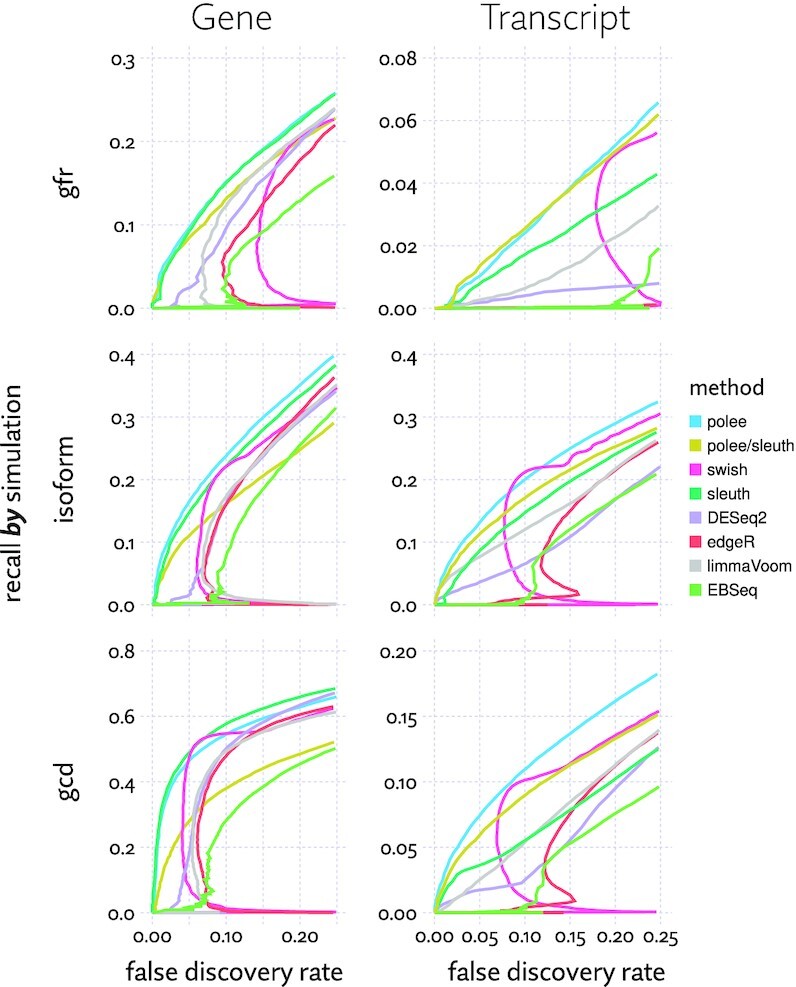
Plots of false discovery rate versus recall calling differential expression on simulated RNA-Seq experiments. False discovery rate (i.e., the proportion of differential expression calls that are incorrect) and recall (i.e. proportion of differentially expressed features identified) were computed for each method on three simulations (gfr, isoform and gcd, explained in the text), with a variety of methods. Each line represents the aggregate FDR-recall curve across 20 replicates of the simulation, each consisting of six samples split across two conditions.

The three simulations, labeled ‘gfr’, ‘isoform’ and ‘gcd’ correspond to three sets of assumptions. The gfr simulation matches simulated effect sizes to those detected by Cufflinks (65) in a reference dataset. The ‘gcd’ simulation adopts the assumption that gene expression is perturbed while holding isoform mixtures fixed, and the ‘isoform’ simulation assumes the expression values of transcripts are perturbed independently of each other. Results from these simulations are shown in Figure 8.

On the column to the right, we see that differential transcript expression tests show both polee and polee/sleuth with significantly improved performance over other methods (i.e. greatly increased recall at the same fdr levels). These results suggest that samples drawn in proportion to the likelihood are more informative than bootstrap samples for this task, and that most informative of all is actually including the likelihood function, or its approximation, in the model. When detecting gene-level differential expression, Polee very slightly trails Sleuth, which exceeds the performance of all the other methods. Oddly, using likelihood approximation samples with gene-level Sleuth analysis (labeled ‘polee/sleuth’ in Figure 8) does not yield similar performance. This may be due to Sleuth’s filtering heuristics being poorly calibrated for samples from the posterior, rather that bootstrap samples.

Swish (28) shows a somewhat unusual precision-recall curve with a high initial false discovery rate, but a very good trade-off at points. Inspecting the results, this appears to be due to it making overconfident differential expression calls for genes or transcripts with high biological variance. Since this simulation involves cases with very high biological variance, and only has three replicates per group, it rewards parametric models of biological variance, and can trip up nonparametric models like Swish. The authors of Swish are aware of this limitation, noting that it does ‘not necessarily have sufficient power to detect differential expression when the sample size is <4 per group’, as is the case here. Despite this, that it matches or outperforms Polee and Sleuth and certain FDR cutoffs further reinforces the importance of accounting for estimation uncertainty as these three methods do.

### Differential expression calls made with approximate likelihood are more internally consistent

Evaluating the accuracy of differential expression calls is fraught by a lack of any agreed upon ground truth. Simulations are one way around that issue, but assume the model of expression and sequencing used to generate the simulated reads is a good approximation of reality. Here we explore another option: calling differential expression with a large number of samples, then testing the ability to recover the same calls with a small number of samples. This avoids putting our faith in the verisimilitude of a simulation, but to be a reasonable proxy for accuracy it instead assumes the model converges to the correct result with enough replicates. Models can of course be both perfectly internally consistent and totally wrong, but taken together with the simulation benchmark in the previous section, consistency makes a case for accuracy in real data.

Using brain tissue data from GTEx (35), we compared the same regression model using five different approaches to transcript quantification: maximum likelihood estimates, maximum likelihood with bootstrap variance estimates, maximum likelihood with variance estimated from a Gibbs sampler, posterior mean estimates generated from the likelihood approximations, and the full approximated likelihood. In addition to running regression with all 13 brain tissues, we also evaluated pairwise differential expression between a transcriptionally similar pair of tissues (hippocampus and amygdala), and a transcriptionally divergent pair (cortex and cerebellum).

**Figure 9.**
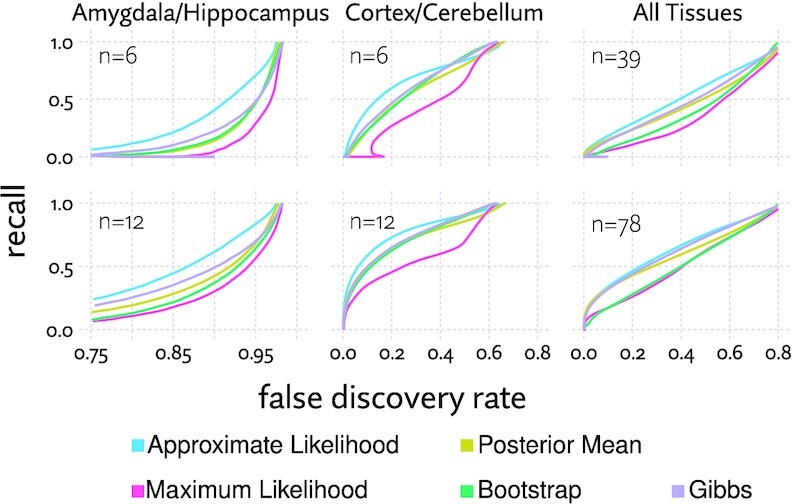
FDR/recall curves for subsets of the GTEx data, using different methods of estimating transcript expression. For each curve, the same regression model is used, with expression modeled as either maximum likelihood or posterior mean point estimates, bootstrap estimates of variance, Gibbs sampler estimates of variance, or approximate likelihood. Note the x-axis has been adjusted for each column to show the details of the curve.

Each run was compared to the same regression model run with a larger number of replicates (96 for the pairwise tests, and 1443 with all tissues). These tests were run with 10 different random subsets to draw the aggregate FDR/recall curves in Figure 9.

The results show broadly that the full approximate likelihood model outperforms point estimates, as well as bootstrap and Gibbs sampler estimates. Estimating variance with a Gibbs sampler is the most competitive alternative, outperforming bootstrap variance estimates. This agrees closely with with the results for ‘polee/sleuth’ we saw in the simulated differential expression benchmark, where we improved the baseline performance of Sleuth by using approximate likelihood samples rather that bootstrap samples. Posterior mean estimates offer an improvement over maximum likelihood estimates, and appear to begin to catch up to the approximate likelihood approach when a large enough number of samples is used. An important caveat is that producing posterior mean point estimates either involves MCMC, or a variational inference, so in generating the estimates, there is little or no performance advantage to using posterior mean estimates instead of approximate likelihood. In the pairwise tests, bootstrap estimates perform similarly to using posterior mean point estimates, while underperforming when using all samples.

The amygdala versus hippocampus test shows very poor performance for all the involved methods, as the differences that do exist between these tissues are small or inconsistent, so they are only reliably detectable with a large number of samples. Nonetheless, approximate likelihood does make better use of the limited data, showing a clear improvement.

### Approximate likelihood improves estimates of pairwise correlation

Large amounts of available sequencing data have led to an increasing emphasis on deciphering the functional relationships between genes. Co-expression networks are often a preliminary step towards inferring regulatory networks (66). Constructing co-expression networks necessitates estimating pairwise correlation or covariance between genes or transcripts across samples. There are many pairs, but relatively few that are highly correlated or highly anticorrelated, so results can easily be contaminated by false positives, a particular risk with low expression genes, and pairs of similar isoforms.

Co-expression in the the GTEx data was examined by Saha *et al.* (67). To control false-positives, aggressive ad hoc filtering was done on the feature set. In addition to including only isoforms with relatively high expression (‘isoforms with at least 10 samples with ≥1 transcripts per million (TPM) and ≥6 reads’), additional filters were applied for isoform variability, mappability, and many features were simply removed to maintain computational tractability. This left only 6000 genes and 9000 isoforms (for comparison, Ensembl annotates nearly 200 000 transcripts). After filtering, a precision matrix was estimated using a graphical lasso model.

This filtering procedure reduces false-positives, but at the cost of potentially introducing false-negatives. A model affording a more principled accounting of estimation uncertainty would obviate the need for much of this ad hoc filtering.

To explore this idea, we used a more simplistic analysis of co-expression, computing pairwise Spearman correlation matrices across all annotated transcripts, with no filtering whatsoever. To see how the choice of estimate affected the results, we did this with maximum likelihood, posterior mean, bootstrap, and approximate likelihood. To avoid division by zero, and to otherwise slightly moderate the effects of zeros, we added a pseudocount of 0.1 TPM to all estimates in the maximum likelihood and bootstrap samples. The uncertainty information provided by bootstrap and approximate likelihood was incorporated by computing the average Spearman correlation across 20 samples.

**Figure 10.**
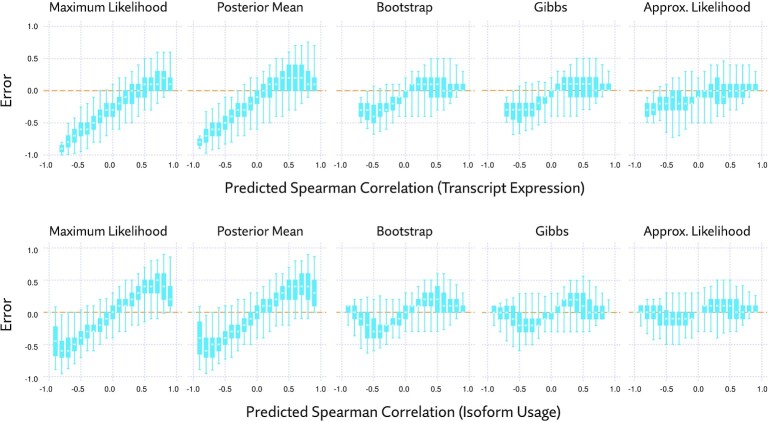
Consistency of Spearman correlation using different methods of estimating transcript expression. Pairwise Spearman correlation was computed for every pair of transcripts using maximum likelihood and posterior mean point estimates, as well as averages across bootstrap samples, Gibbs sampler output, and samples from the approximate likelihood. In the upper plots, transcript expression was used, and in the lower, isoform usage (transcript expression divided by the overall expression of its gene). Error here measures the difference between estimates made using all 118 cortex samples, and estimates using a random subset of 12 samples (i.e. ‘true’ correlation minus predicted correlation). These plots show the aggregate error across ten random subsets. Boxplots are drawn with upper and lower whiskers corresponding to the 99th and 1st percentile, respectively.

As with differential expression, there are no plausible gold standard estimates to compare to, so we resorted to using consistency as a proxy for accuracy. We selected one tissue from the GTEx data, cortex, consisting of 118 samples, and computed the correlation matrices. Treating this as ground truth for each respective estimate, we recomputed the matrices using random subsamples of 12 of the 118 samples, and measured the difference between each element in the matrix. This was repeated 10 times for different random subsamples. The aggregate results are plotted in Figure 10.

Looking first at transcript expression (Figure 10, upper plots), we see that point estimates tend to produce moderately unreliable estimates of positive correlation, and extremely unreliable estimates of negative correlation. In large part, this is remedied by using either bootstrap or Gibbs samples or approximate likelihood, with the latter offering a slight improvement. When considering isoform usage (Figure 10, lower plots), point estimates are highly unreliable when measuring either positive or negative correlation. Bootstrap and Gibbs estimates improve this, but approximate likelihood estimates are clearly the most reliable here.

## DISCUSSION

Here we have described how a full likelihood model of RNA-Seq transcript expression can be made tractable by approximating the likelihood function. Because including the likelihood, rather than relying on point estimates, better accounts for estimation uncertainty, differential expression calls are more reliable. The method we developed to do so, the Pólya tree transformation, is a general purpose approach to approximating sparse mixture models. Though we have confined ourselves here to showing its benefits on a few analyses, there are other possible applications. Other RNA-Seq analyses, like classification, clustering, and dimensionality reduction could benefit from the same approach. It also presents an opportunity of performing isoform level analysis of single-cell RNA-Seq, accounting for the high estimation uncertainty where there are relatively few reads per cell.

This rethinking of RNA-Seq analysis also poses questions about how best to construct models of transcript expression. In our purposely simplistic model of pairwise transcript correlation we made a point of doing no filtering or preprocessing. Sticking to this principle when developing a more sophisticated model of coregulation, while keeping inference tractable, is not trivial. There is also also additional work needed to scale the method to run inference on tens or hundreds of thousands of cells. Though we were able to run a regression model on the GTEx brain data consisting of 1443 samples and over 39 billion reads, this took over two days to run. Scaling the method to tens or hundreds of thousands of samples or cells will require additional engineering, but with out of core methods and faster evaluation of the approximation, it is a goal that is well within reach.

Gelman (68) describes the way in which statistics is sometimes used, either deliberately or otherwise, to transmute randomness into certainty as ‘uncertainty laundering.’ The two-step process often used in RNA-Seq of first estimating, then separately modeling transcript expression can be considered a form of uncertainty laundering, but a form undertaken often out of practical necessity. We believe the method described here, a general approach to reducing the the computational demands of probabilistic RNA-Seq models, is a significant push in the direction of honest accounting of uncertainty.

## DATA AVAILABILITY

The method is implemented in a Julia package available from https://github.com/dcjones/polee

The mouse body map data used for goodness of fit tests is available at accession PRJNA375882. Accession numbers for the data shown in Table 1 are, from top to bottom, SRR453566, SRR030231, SRR065719, SRR023546 and SRR896663.

## Supplementary Material

lqab046_Supplemental_File

## Notes

Present address: Daniel C. Jones, Fred Hutchinson Cancer Research Center, Seattle, WA, USA.
