# Supplementary material for: Polee: RNA-Seq analysis using approximate likelihood
Source: NAR Genom Bioinform. 2021 May 25;3(2):lqab046. doi: 10.1093/nargab/lqab046 (PMC8152449; doi:10.1093/nargab/lqab046)
Supplement: lqab046_Supplemental_File [file lqab046_supplemental_file.pdf]

Supplemental Methods and Materials

April 11, 2021

1 Performance

Approximating the likelihood, evaluating the approximation, and sampling from the approximation are all efficient operations. In Figure 1 we compare the overall time needed to generate samples, comparing Polee to Kallisto. In Figure 2 we compare the reduction in time and space made by our likelihood approximation scheme in comparison to the approximate factorization scheme proposed by Zakeri et al. [2017].

2 Goodness of fit evaluated on more samples

Goodness of fit of the approximation was measured by looking at distributions of p-values in null-hypothesis tests against samples from a Gibbs sampler. In that section we choose one sample to focus on arbitrarily from a mouse body map study [Li et al., 2017], but these distributions of p-values are remarkably consistent across every sample from that experiment, which we show in Figure 3.

3 Properties of the transformation

First, for convenience, we will restate the definition of the Pólya tree transformation. The transformation T from $(u_1, y) \in (\mathbb{R}_+, (0,1)^{n-1})$ to $x \in \mathbb{R}_+^n$ is defined in terms of *intermediate values* $u_1, \ldots, u_{2n-1}$ for each node, which have the following relation,

$$u_{\text{left}(i)} = y_i u_i \tag{1}$$
$$u_{\text{right}(i)} = (1 - y_i) u_i \tag{2}$$

The result of the transformation is then simply the intermediate values from the leaf nodes:

$$x_i = u_{i+n-1}, \text{ for } i = 1, \ldots, n \tag{3}$$

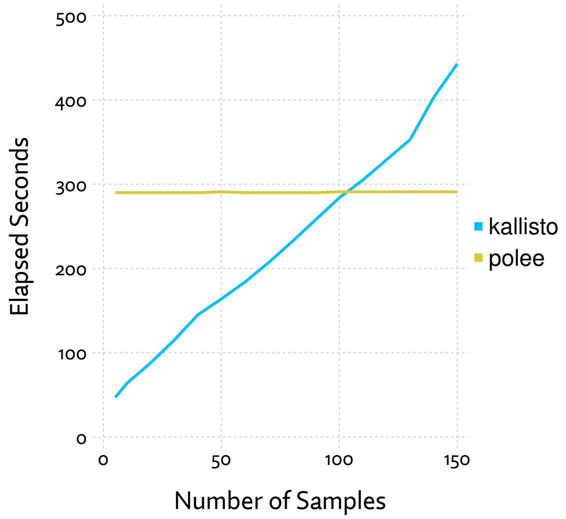

Figure 1: Overall run time to generate samples using Kallisto (bootstrap) or Polee (approximate the likelihood and sample directly from the approximation). Polee requires additional run-time up front to approximate the likelihood function, but once computed, generating samples is exceedingly efficient, with the time only increasing by about 0.69 seconds between 1 sample and 150.

In everything that follows, we adopt the indexing convention that nodes $1, \ldots, n-1$ are internal nodes and $n, \ldots, 2n-1$ leaf nodes. Furthermore, the internal nodes are numbered so that no node has a index smaller than its parent.

Lemma 3.1. *If $leaves(i)$ is the set of indexes of leaf nodes in i's subtree, then*

$$u_i = \sum_{j \in leaves(i)} u_j = \sum_{j \in leaves(i)} x_{j-n+1}$$

Proof. This simply says that each intermediate stick piece is the same length as the sum of the parts it is ultimately broken into, and is easy to see through induction.

Let i be the index of a leaf node. The subtree's only leaf is i, so the theorem is trivially true,

$$u_i = x_{i-n+1} = \sum_{j \in \text{leaves}(i)} x_{j-n+1}$$

Next consider any internal node i where the theorem is true of its children.

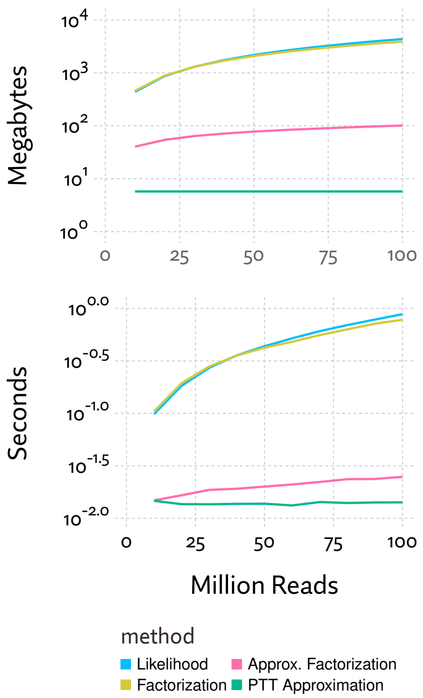

Figure 2: A comparison of the space and time needed to evaluate the likelihood function and its approximations. A GTEx sample (SRR1340508) was subsampled to, and its likelihood evaluated for, increasing numbers of reads. The upper plot shows the memory in megabytes necessary to evaluate the function, the lower plot, the time in seconds necessary to evaluate the function once.

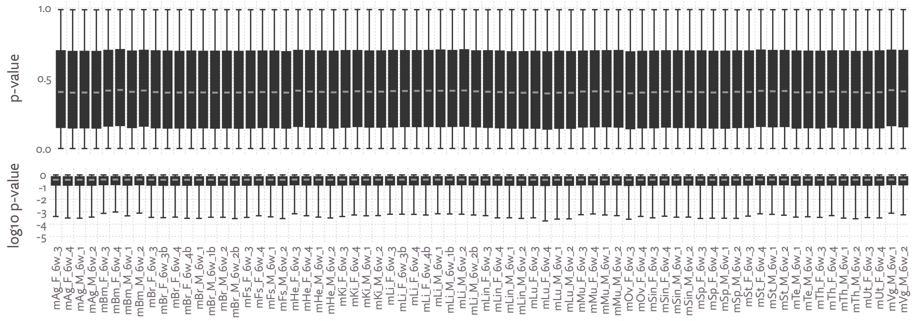

Figure 3: The likelihood function for every sample from [Li et al., 2017] was approximated. 1000 samples were drawn with probability proportional to the approximated likelihood, and 1000 samples were drawn using a Gibbs sampler. A Wilcoxon signed-rank test was performed for each annotated transcript, and the distribution plotted in a boxplot, drawn with upper and lower whiskers corresponding to the 99th and 1st percentile, respectively. An ideal fit would have a uniform distribution of p-values.

That is, where

$$u_{\text{left}(i)} = \sum_{j \in \text{leaves}(\text{left}(i))} x_{j-n+1}$$
$$u_{\text{right}(i)} = \sum_{j \in \text{leaves}(\text{right}(i))} x_{j-n+1}$$

Then,

$$\begin{aligned} u_i &= y_i u_i + (1 - y_i) u_i \\ &= u_{\text{left}(i)} + u_{\text{right}(i)} \\ &= \sum_{j \in \text{leaves}(\text{left}(i))} x_{j-n+1} + \sum_{j \in \text{leaves}(\text{right}(i))} x_{j-n+1} \\ &= \sum_{j \in \text{leaves}(i)} x_{j-n+1} \end{aligned}$$

By mathematical induction, the theorem is true of all $i = 1, \ldots, 2n - 1$. □

Theorem 3.2. *$T : (\mathbb{R}_+, (0,1)^{n-1}) \mapsto \mathbb{R}_+^n$ is a bijection.*

Proof. For any $u_1, u_1' \in \mathbb{R}_+$, and $y, y' \in (0,1)^{n-1}$, where $(u_1, y) \neq (u_1', y')$, let $x = T((u_1, y))$ and $x' = T((u_1', y'))$.

Suppose $u_1 \neq u'_1$, then by Lemma 3.1

$$\sum_{j=1}^{n} x_j = u_1 \neq u'_1 = \sum_{j=1}^{n} x'_j$$

and therefore $x \neq x'$.

Suppose $u_1 = u'_1$, and let i be the index of the first $y_i \neq y'_i$. Since nodes are numbered so that ancestors have smaller indices than their children, for every ancestor j of i, $y_j = y'_j$, therefore $u_i = u'_i$. All intermediate values are positive (this follows from u_1 being positive and elements of y residing on an open $(0, 1)$ interval), therefore

$$u_{\text{left}(i)} = y_i u_i \neq y'_i u_i = y'_i u'_i = u'_{\text{left}(i)}$$

And by Lemma 3.1

$$\sum_{j \in \text{leaves}(\text{left}(i))} x_{j-n+1} \neq \sum_{j \in \text{leaves}(\text{left}(i))} x'_{j-n+1}$$

so $x \neq x'$.

As $x \neq x'$ in either case, T is injective.

To show that T is surjective as well, consider any $x \in \mathbb{R}^n_+$.

Equations 1 and 2 can be rewritten as

$$u_i = u_{\text{left}(i)} + u_{\text{right}(i)} \tag{4}$$

$$y_i = \frac{u_{\text{left}(i)}}{u_{\text{left}(i)} + u_{\text{right}(i)}} \tag{5}$$

First, following Equation 3, let $u_{i+n-1} = x_i$ for $i = 1, \ldots, n$, thus defining leaf node intermediate values. The remaining intermediate values u_i, for $i = 1, \ldots, n-1$ are then defined according to Equation 4, and y_i for $i = 1, \ldots, n$ according to Equation 5.

Importantly, since elements of x are positive, it follows that all intermediate values are positive, so the denominator of Equation 5 is never zero and $y_i \in (0, 1)$.

As Equations 1, 2 are satisfied if Equations 4, 5 are, and u_1, y are defined by the latter, it follows that $T((u_1, y)) = x$ and T is surjective, and thus bijective. □

Corollary 3.3. *If* $u_1 = 1$ *is fixed, then the resulting transformation is a bijection between* $(0, 1)^{n-1}$ *and* $\Delta^{n-1} = \{x \in \mathbb{R}^n_+ | \sum_{i=1}^{n} x_i = 1\}$.

Proof. A restriction of a bijection remains a bijection so we need only show that the image is Δ^{n-1}. It follows directly from Lemma 3.1 that the codomain of the restriction is Δ^{n-1}. Furthermore, any $x \in \Delta^{n-1}$ has a (u_1, y) where $T((u_1, y)) = x$, and again by Lemma 3.1, $u_1 = 1$, so (u_1, y) is in the restricted domain, and thus the image is Δ^{n-1}. □

For the purposes of approximating the RNA-Seq likelihood function, we only care about this special case mapping onto the simplex.

For the approximation to be useful for computing probability densities of transformed variables, the determinant of the Jacobian must also be efficiently computable, which we show here.

Theorem 3.4. *Let* $(u_1, y) \in (\mathbb{R}_+, (0,1)^{n-1})$*, and* J_T *be the Jacobian matrix of* $T((u_1, y))$*. Further, let* u_i *for* $i = 2, \ldots, 2n-1$ *be the intermediate values as defined in Equations 1 and 2. Then*

$$|\det(J_T)| = \prod_{i=1}^{n-1} u_i$$

Or in words, the absolute value of the Jacobian determinant is simply the product of the internal node intermediate values.

Proof. One way to derive the determinant for J_T is to separate the transformation T into the composition of a number of simpler transformations. The stick breaking metaphor suggests as natural decomposition: each break can be treated as its own transformation. If T is thought of as a sequence of $n-1$ breaks, it can be represented as

$$T = T_{n-1} \circ \cdots \circ T_1$$

where T_i is the ith break and has the associated break proportion y_i. This would let us write

$$|\det(J_T)| = \prod_{i=1}^{n-1} |\det J_{T_i}|$$

where J_{T_i} is the Jacobian matrix for T_i.

The single break transformation

$$T_i : (\mathbb{R}_+^i, (0,1)^{n-i}) \mapsto (\mathbb{R}_+^{i+1}, (0,1)^{n-i-1})$$

can be thought of as taking i sticks lengths, and $n-i$ remaining breaking proportions, and breaking one of the sticks according to the first remaining proportion. Equivalently, T_i reflects taking one step in the tree traversal, computing $u_{\text{left}(i)}, u_{\text{right}(i)}$ from u_i, y_i, and then discarding these latter two values.

Incidentally, because only two values are replaced, it is not hard to see that each of these transformations is also a bijection between different n dimensional spaces.

To give a concrete example of T being decomposed into single break transformations, consider a balanced tree with $n = 4$ leaves, and the input $u_1 = 100, y = (0.2, 0.6, 0.9)$. In the following table, each row gives the input to T_i, and the subsequent row its output. The final result is a vector in $\mathbb{R}_+^n$.

Because T_i only replaces u_i, y_i with $u_{\text{left}(i)}, u_{\text{right}(i)}$, the Jacobian matrix is the n by n identity matrix except for a 2 by 2 submatrix so that,

i	$\mathbb{R}_+^i$	$(0,1)^{n-i}$
1	100	$0.2, 0.6, 0.9$
2	$20, 80$	$0.6, 0.9$
3	$12, 8, 80$	0.9
	$12, 8, 72, 8$	

$$
\begin{aligned}
|\det(J_{T_i})| &= \left\| \begin{array}{cc} \frac{\partial u_{\text{left}(i)}}{\partial u_i} & \frac{\partial u_{\text{left}(i)}}{\partial y_i} \\ \frac{\partial u_{\text{right}(i)}}{\partial u_i} & \frac{\partial u_{\text{right}(i)}}{\partial y_i} \end{array} \right\| \\
&= \left\| \begin{array}{cc} y_i & u_i \\ 1 - y_i & -u_i \end{array} \right\| \\
&= |-y_i u_i + y_i u_i - u_i| \\
&= u_i
\end{aligned}
$$

And therefore

$$
|\det(J_T)| = \prod_{i=1}^{n-1} |\det J_{T_i}| = \prod_{i=1}^{n-1} u_i
$$

□

A Dirichlet process is a special case of a Pólya tree distribution [Ferguson, 1974]. Here we show a variation of this fact, that a Dirichlet distribution can be represented using any Pólya tree transformation, regardless of tree topology, if appropriately applied to Beta distributed random variables.

This is important to RNA-Seq because a multinomial distribution is often assumed when there is thought to be no read ambiguity. A multinomial distribution renormalized to form a distribution over probability vectors is a Dirichlet distribution. So this theorem says that if Pólya tree transformed Beta random variables are fit to the multinomial likelihood, nothing is lost except for a constant of proportionality. If the underlying distributions can reasonably mimic Beta distributions, then we can expect the fit to the multinomial to be very good.

Definition 3.5 (Hierarchical Beta distribution family)**.** *For $n > 1$, let $T : (0,1)^{n-1} \mapsto \Delta^{n-1}$ be a Pólya tree transformation and $\alpha \in \mathbb{R}_+^n$.*

For $i \in \{1, \dots, n-1\}$, let

$$
a_i = \sum_{j \in \mathit{leaves}(i)} \alpha_{j-n+1}
$$

where $\mathit{leaves}(\cdot)$ corresponds to T's tree topology. Put in words, if we associate α_j with the jth leaf node (which is node $j + n - 1$ in our numbering scheme), a_i is the sum of α entries corresponding to the leaf node descendants of internal node i.

A random variable X is HierarchicalBeta(T, α) distributed iff $X \sim T(Y)$, where $Y = (Y_1, \ldots, Y_{n-1})$ are independent random variables with

$$Y_i \sim Beta(a_{left(i)}, a_{right(i)})$$

Hierarchical Beta is simply the family of distributions induced by applying a Pólya tree transformation to Beta distributed stick breaking proportions with a specifically chosen parameterization scheme. The larger distribution family formed without constraining the Beta parameters is discussed by Dennis [1991], who call it the the "hyper-Dirichlet type 1" distribution. Recall that a Pólya tree distribution is a (potentially infinite) hierarchical stick breaking process [Lavine, 1992, 1994, Mauldin et al., 1992] with Beta distributed breaks. Hierarchical Beta distributions are thus a specific subset of Pólya tree distributions.

Definition 3.6. *A* terminal stick break *transformation*

$$B_{ijk} : (\mathbb{R}_+^{n-1}, (0,1)) \mapsto \mathbb{R}_+^n$$

for $1 \leq i \leq n-1$ and $1 \leq j < k \leq n$ is defined by

$$x' = B_{ijk}(x, y)$$

where x' is formed from x by removing x_i and inserting yx_i and $(1-y)x_i$ such that they are x'_j and x'_k, respectively.

This is a bijection, and the inverse B_{ijk}^{-1}, we call an initial stick mend*. It removes x'_j and x'_k and inserts $x_i = x'_j + x'_k$, and additionally yields $y = \frac{x'_j}{x'_j + x'_k}$.*

In the proof of Theorem 3.4 we described how a Pólya tree transformation can be thought of as a series of single stick break transformations, which we used to derive the Jacobian determinant. In most trees there is a choice in the order of single stick breaks, equivalent to choosing one of multiple possible pre-order traversals of the tree, each of which results in a different representation of the same Pólya tree transformation. Regardless of the ordering, there is always some final break, and this break is a terminal stick break B_{ijk} for some i, j, k.

Some notation will be useful to decompose and build up Pólya tree distributions.

Definition 3.7. *If*

$$T_i : (\mathbb{R}_+^j, (0,1)^k) \mapsto (\mathbb{R}_+^{j+1}, (0,1)^{k-1})$$

is a single stick break transformation, as described in the proof for Theorem 3.4, which produces one more stick length by using the first breaking proportion, and if $k > 1$, we can drop the last unused breaking proportion to form a new transformation we denote

$$T_i^- : (\mathbb{R}_+^j, (0,1)^{k-1}) \mapsto (\mathbb{R}_+^{j+1}, (0,1)^{k-2})$$

Similarly, we can add another unused breaking proportion if $k \geq 0$, which we'll write as

$$T_i^+ : (\mathbb{R}_+^j, (0,1)^{k+1}) \mapsto (\mathbb{R}_+^{j+1}, (0,1)^k)$$

This notation lets us peel off the final break of a Pólya tree transformation. As we saw, the last break is a terminal stick break B_{ijk} for some i, j, k. So any Pólya tree transformation T with $n > 2$ can be written as $B_{ijk} \circ S$, for a transformation

$$S : (\mathbb{R}_+, (0,1)^{n-1}) \mapsto (\mathbb{R}_+^{n-1}, (0,1))$$

If we dispense with S's unused breaking proportion, we have

$$S^- : (\mathbb{R}_+, (0,1)^{n-2}) \mapsto \mathbb{R}_+^{n-1}$$

which is itself a Pólya tree transformation. Similarly, we can add another breaking proportion with the T^+ notation to extend a Pólya tree transformation.

Lemma 3.8 (Hierarchical Beta breaking)**.** *Let $X \sim$ HierarchicalBeta(T, α), where T is a Pólya tree transformation. Let $\alpha \in \mathbb{R}_+^n$, and $Z \sim$ Beta(a, b) where $a + b = \alpha_i$ for some $1 \leq i \leq n$, and let B_{ijk} be a terminal stick break, then*

$$B_{ijk}(X, Z) \sim \textit{HierarchicalBeta}(B_{ijk} \circ T^+, \alpha')$$

where α' is formed by removing α_i from α and inserting a and b such that $\alpha'_j = a$ and $\alpha'_k = b$.

Proof. The original Hierarchical Beta distribution is defined by the Pólya tree transformation T applied to $n-1$ Beta distributed random variables $Y_1, \ldots, Y_{n-1}$, parameterized with α as they are in Definition 3.5.

Applying the terminal stick break B_{ijk} simply does one more break according to one more Beta distributed random variable Z. So the resulting distribution is defined by the the Pólya tree transformation $B_{ijk} \circ T^+$ applied to Beta random variables $Y' = (Y_1, \ldots, Y_{n-1}, Z)$. It only remains to be seen that Y' follows the Hierarchical Beta parameterizations with α'.

Z is defined to trivially obey these rules, and restricting $a + b = \alpha_i$ and inserting a, b into α to form α' preserves the parameterization of the rest of the distribution, so we can say $B_{ijk}(X, Z) \sim \text{HierarchicalBeta}(B_{ijk} \circ T^+, \alpha')$. □

Lemma 3.9 (Dirichlet mending)**.** *If $X \sim$ Dirichlet(α) and B_{ijk}^{-1} is an initial stick mend, then*

$$B_{ijk}^{-1}(X) \sim (\textit{Dirichlet}(\alpha'), \textit{Beta}(\alpha_j, \alpha_k))$$

where α' is formed from α by removing α_j and α_k and inserting their sum so that $\alpha'_i = \alpha_j + \alpha_k$.

Proof. This follows from two properties of the Dirichlet distribution: subcompositional invariance and amalgamation invariance (see, for example, Pawlowsky-Glahn et al. [2015] p. 121). If $x \sim \text{Dirichlet}(\alpha)$, the former property says, in the narrow case needed here, that for any $1 \leq k_1 < k_2 \leq n$,

$$\left(\frac{x_{k_1}}{x_{k_1} + x_{k_2}}, \frac{x_{k_2}}{x_{k_1} + x_{k_2}} \right) \sim \text{Dirichlet}(\alpha_{k_1}, \alpha_{k_2})$$

or, equivalently,

$$\frac{x_{k_1}}{x_{k_1} + x_{k_2}} \sim \text{Beta}(\alpha_{k_1}, \alpha_{k_2})$$

The latter property says that if $\{1, \ldots, n\}$ is partitioned into $k > 1$ nonempty sets $I_1, \ldots, I_k$, then

$$\left(\sum_{i \in I_1} x_i, \ldots, \sum_{i \in I_k} x_i \right) \sim \text{Dirichlet} \left(\sum_{i \in I_1} \alpha_i, \ldots, \sum_{i \in I_k} \alpha_i \right)$$

For any $X \sim \text{Dirichlet}(\alpha)$, and initial stick mend B_{ijk}^{-1}, let $(X', Y) = B_{ijk}^{-1}(X)$.

From the definition of an initial stick mend, $Y = \frac{X_j}{X_j + X_k}$. The subcompositional invariance property then gives us

$$Y \sim \text{Beta}(\alpha_j, \alpha_k)$$

Since α' is formed by by summing two elements of α, by the amalgamation invariance property,

$$X' \sim \text{Dirichlet}(\alpha')$$

□

Theorem 3.10. *For any Pólya tree transformation* $T : (0, 1)^{n-1} \mapsto \Delta^{n-1}$ *where* $n \geq 2$ *and any* $\alpha \in \mathbb{R}_+^n$

$$\textit{Dirichlet}(\alpha) = \textit{HierarchicalBeta}(T, \alpha)$$

In other words, the Dirichlet distribution family and the Hierarchical Beta distribution family are exactly the same, regardless of the tree topology used for the Hierarchical Beta.

Proof. The proof will proceed by induction over n.

(Base case) Let $n = 2$. For any $\alpha \in \mathbb{R}_+^2$ let $Y \sim \text{Beta}(\alpha_1, \alpha_2)$. Note that, because Dirichlet generalizes Beta, $(Y, (1 - Y)) \sim \text{Dirichlet}(\alpha)$.

There is only one Pólya tree transformation $T : (0, 1) \mapsto \Delta^1$, which takes the form $T(Y) = (Y, 1 - Y)$ and by Definition 3.6, $T(Y) = (Y, 1 - Y) \sim \text{HierarchicalBeta}(T, \alpha)$, so we have

$$\text{Dirichlet}(\alpha) = \text{HierarchicalBeta}(T, \alpha)$$

(Inductive step) Now assume the proposition is true for some $n \geq 2$.

For any $\alpha \in \mathbb{R}_+^{n+1}$ and any Pólya tree transformation $T : (0, 1)^n \mapsto \Delta^n$, we can decompose T as $T = B_{ijk} \circ S$ for transformations S and B_{ijk} where B_{ijk} is a terminal stick break for some indexes i, j, k.

By Lemma 3.9, if $X \sim \text{Dirichlet}(\alpha)$ then

$$(X', Y) = B_{ijk}^{-1}(X) \sim (\text{Dirichlet}(\alpha'), \text{Beta}(\alpha_i, \alpha_j))$$

where α' is defined as it is in Lemma 3.9.

By the inductive hypothesis, X' is also Hierarchical Beta distributed for any Pólya tree transformation onto Δ^{n-1}, and since S^- is a Pólya tree transformation onto Δ^{n-1},

$$\text{Dirichlet}(\alpha') = \text{HierarchicalBeta}(S^-, \alpha')$$

Because B_{ijk} and B_{ijk}^{-1} are inverses,

$$B_{ijk}(X', Y) \sim \text{Dirichlet}(\alpha)$$

and by Lemma 3.8,

$$B_{ijk}(X', Y) \sim \text{HierarchicalBeta}(T, \alpha)$$

and so we have

$$\text{Dirichlet}(\alpha) = \text{HierarchicalBeta}(T, \alpha)$$

And by induction, this equality holds for all such α, T and any $n \geq 2$. □

This result can be strengthened to say that not only can any Dirichlet distribution be constructed using any Pólya tree transformation of the same dimensionality, but the Hierarchical Beta construction is the only way to do so. First, we need a simple result about random variables under bijections.

Lemma 3.11. *For any random variables $Y = (Y_1, \ldots, Y_n)$ and $Y' = (Y'_1, \ldots, Y'_n)$, and bijection T, if $T(Y)$ and $T(Y')$ are identically distributed, Y and Y' must also be identically distributed.*

Proof. To show the contrapositive, suppose $T(Y)$ and $T(Y')$ are not identically distributed. There is then some y where $P(T(Y) = y) \neq P(T(Y') = y)$. Since

$$P(T(Y) = y) = |\det J_T| P(Y = T^{-1}(y))$$

and

$$P(T(Y') = y) = |\det J_T| P(Y' = T^{-1}(y))$$

where J_T is the Jacobian matrix for T, it follows that

$$|\det J_T| P(Y = T^{-1}(y)) \neq |\det J_T| P(Y' = T^{-1}(y))$$
$$P(Y = T^{-1}(y)) \neq P(Y' = T^{-1}(y))$$

Therefore Y and Y' are not identically distributed either. □

Next, we can strengthen Theorem 3.10.

Corollary 3.12. *For any Pólya tree transformation T, $\alpha \in \mathbb{R}^n_+$, and random variables $Y = (Y_1, \ldots, Y_{n-1})$. If*

$$T(Y) \sim \textit{Dirichlet}(\alpha)$$

then

$$Y_i \sim \textit{Beta}(a_{\textit{left}(i)}, a_{\textit{right}(i)})$$

for $i \in \{1, \ldots, n-1\}$ where a is defined as it is in Definition 3.5.

Proof. For any Pólya tree transformation T and $\alpha \in \mathbb{R}^n$, we know from Theorem 3.10, that if $Y = (Y_1, \ldots, Y_n)$ are Beta distributed with the Hierarchical Beta parameterization, then

$$T(Y) \sim \text{Dirichlet}(\alpha)$$

From Lemma 3.11, any other random variables $Y' = (Y'_1, \ldots, Y'_n)$ where $T(Y') \sim \text{Dirichlet}(\alpha)$ must be identically distributed to Y, and so must also be Beta distributed with the same parameterization. □

To summarize these results: any Dirichlet distribution can be constructed using any Pólya tree transformation of the same dimensionality, and furthermore this construction is unique.

4 Minimizing KL-divergence

Minimizing the KL-divergence between the approximation q and the normalized likelihood function $\mathcal{P}(r|\cdot)$ (defined in Equation 3 the Methods section), with a set of observed reads r, uses a standard variational inference procedure, which we will briefly recapitulate here.

Recall that the approximation q can be represented as a parameterized transformation S applied to a non-parameterized distribution (i.e., the reparameterization trick). In our case, the non-parameterized distribution is simply a standard $n-1$ dimensional Normal distribution $N(0, I)$. The transformation S includes the sinh-asinh transformation, shifting and scaling, the logistic transformation, and the Pólya tree transformation T, which we collapse into a single function here for simplicity.

Explicitly, the combined transformation, for $y \in \mathbb{R}^{n-1}$ is

$$S_{\mu,\sigma,\alpha}(y) = T(\text{logistic}(\mu + \sigma \odot \sinh(\text{asinh}(y) + \alpha)))$$

where μ, σ, α is the parameterization, $\odot$ is the element-wise product, and the logistic, sinh, and asinh functions are also applied element-wise. The logistic function has the standard definition $\text{logistic}(x) = (1 + \exp(-x))^{-1}$, and T is a Pólya tree transformation. Note that T is not parameterized, since it is chosen prior to optimization, as discussed in in the Methods section. To simplify notation, we will combine parameter vectors as $\phi = (\mu, \sigma, \alpha)$ and use S_ϕ to denote the parameterized transformation.

The approximate normalized likelihood q_ϕ is defined as the transformation S_ϕ applied to a multivariate standard-normal $z \sim \text{Normal}(0, I)$. If p is the density function for this Normal distribution, the approximation's density function is then

$$q_\phi(x) = p(S_\phi^{-1}(x))|\det J_{S_\phi^{-1}}|$$

where $J_{S_\phi^{-1}}$ is the Jacobian matrix for the inverse S_ϕ transformation.

With this set up in mind, the objective function can be rewritten in a form amenable to stochastic gradient descent.

$$
\begin{aligned}
&\arg\min_{\phi} \mathrm{KL}(q_\phi || \mathcal{P}) \\
&= \arg\min_{\phi} E_{x\sim q_\phi}\left[\log q_\phi(x) - \log \mathcal{P}(r|x)\right] \\
&= \arg\max_{\phi} E_{x\sim q_\phi}\left[\log \mathcal{P}(r|x) - \log q_\phi(x)\right] \\
&= \arg\max_{\phi} E_{x\sim q_\phi}\left[\log \mathcal{P}(r|x) - \log p(S_\phi^{-1}(x)) - \log|\det J_{S_\phi^{-1}}|\right] \\
&= \arg\max_{\phi} E_{z\sim N(0,I)}\left[\log \mathcal{P}(r|S_\phi(z)) - \log p(z) + \log|\det J_{S_\phi}|\right] \\
&= \arg\max_{\phi} E_{z\sim N(0,I)}\left[\log \mathcal{P}(r|S_\phi(z)) + \log|\det J_{S_\phi}|\right] \\
&= \arg\max_{\phi} E_{z\sim N(0,I)}\left[\log P(r|S_\phi(z)) + \log|\det J_{S_\phi}|\right]
\end{aligned}
$$

The penultimate step follows because $E_z[-\log p(z)]$ is a constant (specifically, the entropy of a multivariate standard Normal distribution), and in the final step we replace the normalized likelihood $\mathcal{P}(\cdot)$ with the actual likelihood $P(r|\cdot)$, since the constant of proportionality becomes an irrelevant additive constant here.

Intuitively, the objective is choosing an approximation that maximizes the expected log-likelihood, with a Jacobian term accounting for the transformation. What we are left with is an expectation than can be easily optimized through stochastic gradient descent. Each iteration of the optimization algorithm draws from $N(0, I)$, transforms the vector, evaluates the RNA-Seq log-likelihood function, and adds to that the log of the Jacobian determinant of the transformation, computing the gradients with respect to ϕ. In practice we use Adam Kingma and Ba [2014] to update ϕ estimates each iteration, and 500 iterations, with each iteration estimating gradients using 6 Monte Carlo samples of z.

5 Regression Model

Differential expression is determined throughout the paper using a probabilistic linear regression model on log-expression, but with the additional layer of approximate likelihood.

In defining the model we will use the following notation:

m	number of samples in the experiment
n	number of transcripts
k	number of factors/columns in the design matrix
X	m by n matrix of log-expression values
E	m by n noise matrix
A	m by k design matrix
W	k by n matrix of regression coefficients
μ	length n log-expression bias vector
σ	length n transcript standard deviation vector
$r^{(j)}$	some representation of the RNA-Seq reads for the jth sample
$\mathbf{1}$	ones vector (length m in all uses below)

The basic regression model is defined by,

$$\begin{aligned} E_{ij} &\sim \text{Normal}(0, \sigma_j) \\ X &= \mathbf{1}\mu^T + AW + E \\ r^{(j)}|X &\sim \text{ApproximateLikelihood}(\text{softmax}(X_j^T)) \end{aligned}$$

where X_j^T is the jth row of X, transposed.

The softmax functions transforms the log-expression to relative expression. There are some subtleties of this move noted below. The bias vector μ has a Normal prior with a large variance.

Apart from the inclusion of the extra layer $r|X$, treating the reads as observed and expression values as unobserved, this is a fairly standard log-linear model of gene expression, similar to the one described by Smyth [2004], for example.

Prior on regression coefficients

Operating under the prior expectation that most transcripts are not differentially expressed, W is given a sparsity inducing prior. Elements of W_{ij} have the prior given by,

$$\begin{aligned} W_{ij}|\lambda_{ij} &\sim \text{Normal}(0, \lambda_{ij}) \\ \lambda_{ij}|\eta_{ij} &\sim \text{HalfCauchy}(0, \eta_{ij}) \\ \eta_{ij} &\sim \text{HalfCauchy}(0, 1) \end{aligned}$$

This is a version of what Bhadra et al. [2017] describe as the "Horseshoe+" prior.

Modeling variance

Transcript expression standard deviation σ is shrunk towards standard deviation estimates of transcripts with similar expression.

To do this we choose $h = 15$ "knots" at expression values, equally spaced between the minimum and maximum, and compute weights $u_{j\ell}$ between every transcript j and knot ℓ, using the squared difference between the knot's expression value and the transcript expression averaged across samples. These weights are pre-computed using initial approximate posterior mean expression estimates, obtained by applying the approximation's transformation to a zero vector. Each knot has associated parameters α_ℓ, β_ℓ.

$$\alpha_\ell \sim \text{HalfCauchy}(0, 1)$$
$$\beta_\ell \sim \text{HalfCauchy}(0, 1)$$

Per-transcript standard deviation is Inverse-Gamma distributed with parameters computed as weighted sums of these.

$$\sigma_j \sim \text{InverseGamma}\left(\sum_{\ell=1}^{h} u_{j\ell}\alpha_\ell, \sum_{\ell=1}^{h} u_{j\ell}\beta_\ell\right)$$

Scale penalization

Because RNA-Seq measures relative expression, detecting changes is absolute expression involves additional assumptions. Various normalization schemes have been proposed to overcome this [Bullard et al., 2010]. Other methods avoid the problem by looking for compositional changes [McGee et al., 2019]. Similar to the normalization schemes, our model attempts to find changes in absolute expression by assuming most transcripts or genes are maintaining relatively constant expression (or constant lack of expression).

Because of the the softmax transformation, log-expression values are not on a fixed scale, which is to say for a given sample i, $\sum_{j=1}^{n} \exp(x_{ij})$ is not constrained to sum to any particular number. How relative scales between samples are inferred determines how changes in transcript composition are interpreted as changes in expression.

Because of the sparsity inducing prior, higher probability is achieved when fewer transcripts are differentially expressed. If this assumption is reasonable relative scaling will be arrived at automatically so as to minimize differences between samples. Of course, there are circumstances when this assumption fails, particularly in the presence of large changes in the overall quantity of RNA between groups of cells.

In practice we found it helpful for inference to additionally penalize these exponent sums from straying too far. To find reasonable values, we use initial posterior mean estimates to estimate relative scales that minimize differential expression of highly expressed transcripts, then include an additional Normal

prior in the model on these exponent sums, centered on the scale estimates. This is simply one approach to putting an informative prior on relative scale.

Inference

The model is fit using variational inference implemented in TensorFlow. Computing the Pólya tree transformation efficiently, particularly many different transformations with different tree topologies, using tensor operations is not straightforward. To avoid this issue, we implemented a custom TensorFlow operation in C++ which computes transformations in parallel on CPUs. Currently, there is no GPU version of this operation, so while running inference on GPUs greatly speeds it up (for example, running a regression on 96 samples took 44 minutes with a GPU, and 168 minutes without), computing the Pólya tree transformation remains the bottleneck, and an obvious target for further improvements in efficiency.

When calling differential expression with this model, effect size is always considered explicitly. Because the model is continuous, the posterior probability of no change in expression is zero. We instead look for transcripts or genes with a sufficiently large posterior probability of the effect size being above some threshold. Unlike most null-hypothesis test models which typically (though, not necessarily) adopt a null hypothesis of zero change, there is an extra threshold that must be chosen: the minimum effect size. This is a slight complication, but has the advantage of being up front about what is considered a potentially interesting effect.

6 Bias correction

Bias correction methods implemented in Polee follow a methodology similar our prior work in Jones et al. [2012], but incorporating some of the advancements made in Love et al. [2016] to produce a more complete model. The essential approach is to train a probabilistic classifier to discriminate between observed fragments and random fragments from the same transcripts, then using the resulting probability ratios to compute bias.

1. Randomly sample a subset of reads.
2. Assign these reads to transcripts by maximum likelihood, to produce a set of implied fragments F_f
3. Reposition fragments in F_f uniformly at random within their transcripts to produce a set of hypothetical background fragments F_b.
4. Train a probabilistic classifier on $F_f \cup F_b$ to compute $P(f \in F_f | c(f))$, where $c(f)$ is the confounding features (e.g., GC content) of the fragment.
5. Compute bias for a fragment f as
$$b(f) = \frac{P(c(f)|f \in F_f)}{P(c(f)|f \in F_b)}$$

Here $|F_f| = |F_b|$, so the obvious prior is $P(f \in F_f) = 0.5$, and in such case, the posterior probability is simple to compute from the Bayes factor, or vice versa,

$$P(f \in F_f|c(f)) = \frac{b(f)}{1 + b(f)}$$

$$b(f) = \frac{P(f \in F_f|c(f))}{1 - P(f \in F_f|c(f))}$$

If there are no technical effects biasing read coverage, this classifier will (setting aside any issues of overfitting or noise) match the prior of 0.5 and the bias will be computed as 1 for every fragment.

We train separate models for sequence bias at fragment ends, fragment GC content bias, and positional bias in sequence, at each step reweighting each training example according the classification error of the combined model up to that point in a manner similar to AdaBoost [Bishop, 2006]. Each model is alone is relatively simple.

- **Fragment sequence end** Sequence probability is modeled with a variable-order Markov chain model. The order at each position is fit by a greedy hill climbing approach. At each iteration, the order of the chain is incremented at the position that most improves the cross entropy. Iteration stops when no improvement is possible. Separate models are build for the 3' and 5' ends of the fragment, which correspond to first- and second-strand cDNA synthesis, as we observe slightly different bias patterns for the two sides.

- **Fragment GC content** A simple histogram is used where examples are split into bins so that the number of examples in each bin is approximately equal. The number of bins is optimized over.

- **Positional bias** Fragments are binned by relative position and transcript length, a histogram is computed, and bins are linearly interpolated (and extrapolated). The number of transcript length bins and relative position bins are optimized over.

The optimization carried out in each sub-model is performed by evaluating cross entropy on a portion of the training data that is set aside. The remainder of the training data is used for setting parameters to their maximum likelihood value, which is trivial for histograms and Markov chain models.

References

Anindya Bhadra, Jyotishka Datta, Nicholas G Polson, and Brandon Willard. The horseshoe+ estimator of Ultra-Sparse signals. *Bayesian Anal.*, 12(4): 1105–1131, December 2017.

Christopher M Bishop. Combining models. In *Pattern Recognition and Machine Learning*. springer, 2006.

James H Bullard, Elizabeth Purdom, Kasper D Hansen, and Sandrine Dudoit. Evaluation of statistical methods for normalization and differential expression in mRNA-Seq experiments. *BMC Bioinformatics*, 11:94, February 2010.

Samuel Y Dennis. On the Hyper-Dirichlet type 1 and Hyper-Liouville distributions. *Communications in Statistics - Theory and Methods*, 20(12):4069–4081, January 1991.

Thomas S Ferguson. Prior distributions on spaces of probability measures. *Ann. Stat.*, 2(4):615–629, 1974.

Daniel C Jones, Walter L Ruzzo, Xinxia Peng, and Michael G Katze. A new approach to bias correction in RNA-Seq. *Bioinformatics*, 28(7):921–928, April 2012.

D P Kingma and J Ba. Adam: A method for stochastic optimization. *arXiv preprint arXiv:1412.6980*, 2014.

Michael Lavine. Some aspects of Polya tree distributions for statistical modelling. *Ann. Stat.*, 20(3):1222–1235, 1992.

Michael Lavine. More aspects of Polya tree distributions for statistical modelling. *Ann. Stat.*, 22(3):1161–1176, 1994.

Bin Li, Tao Qing, Jinhang Zhu, Zhuo Wen, Ying Yu, Ryutaro Fukumura, Yuanting Zheng, Yoichi Gondo, and Leming Shi. A comprehensive mouse transcriptomic BodyMap across 17 tissues by RNA-seq. *Sci. Rep.*, 7(1):4200, June 2017.

Michael I Love, John B Hogenesch, and Rafael A Irizarry. Modeling of RNA-seq fragment sequence bias reduces systematic errors in transcript abundance estimation. *Nat. Biotechnol.*, 34(12):1287–1291, December 2016.

R Daniel Mauldin, William D Sudderth, and Stanley C Williams. Polya trees and random distributions. *Ann. Stat.*, 20(3):1203–1221, September 1992.

Warren A McGee, Harold Pimentel, Lior Pachter, and Jane Y Wu. Compositional data analysis is necessary for simulating and analyzing RNA-Seq data. *bioRxiv*, page 564955, March 2019.

Vera Pawlowsky-Glahn, Juan José Egozcue, and Raimon Tolosana-Delgado. *Modeling and Analysis of Compositional Data*. John Wiley & Sons, February 2015.

Gordon K Smyth. Linear models and empirical bayes methods for assessing differential expression in microarray experiments. *Stat. Appl. Genet. Mol. Biol.*, 3(1), February 2004.

Mohsen Zakeri, Avi Srivastava, Fatemeh Almodaresi, and Rob Patro. Improved data-driven likelihood factorizations for transcript abundance estimation. *Bioinformatics*, 33(14):i142–i151, July 2017.
